# Research Progress on Porous Carbon-Based Non-Precious Metal Electrocatalysts

**DOI:** 10.3390/ma16083283

**Published:** 2023-04-21

**Authors:** Hongda Yu, Luming Wu, Baoxia Ni, Tiehong Chen

**Affiliations:** Smart Sensing Interdisciplinary Science Center, Key Laboratory of Advanced Energy Materials Chemistry (MOE), Institute of New Catalytic Materials Science, School of Materials Science and Engineering, Nankai University, Tianjin 300350, China

**Keywords:** oxygen reduction reaction, electrochemical energy, non-precious metal nanoparticles electrocatalysis, porous carbon-based materials

## Abstract

The development of efficient, stable, and economic electrocatalysts are key to the large-scale application of electrochemical energy conversion. Porous carbon-based non-precious metal electrocatalysts are considered to be the most promising materials to replace Pt-based catalysts, which are limited in large-scale applications due to high costs. Because of its high specific surface area and easily regulated structure, a porous carbon matrix is conducive to the dispersion of active sites and mass transfer, showing great potential in electrocatalysis. This review will focus on porous carbon-based non-precious metal electrocatalysts and summarize their new progress, focusing on the synthesis and design of porous carbon matrix, metal-free carbon-based catalysts, non-previous metal monatomic carbon-based catalyst, and non-precious metal nanoparticle carbon-based catalysts. In addition, current challenges and future trends will be discussed for better development of porous carbon-based non-precious metal electrocatalysts.

## 1. Introduction

Industrial development dependent on fossil energy has brought rapid growth to the global economy, but at the same time, there have been serious energy shortages and environmental pollution problems [1]. The development of renewable and sustainable clean energy has become the main research topic [2,3]. Clean energy mainly includes solar energy, wind energy, biological energy, and electrochemical energy, among which electrochemical energy has attracted wide attention due to its high energy conversion efficiency [4]. Electrochemical storage and conversion devices mainly include metal–air cells, fuel cells, and electrolytic cells [5]. These devices realize the conversion between electric energy and chemical energy through an electrochemical reaction, which involves Oxygen Reduction Reaction (ORR), Oxygen Evolution Reaction (OER), Hydrogen Evolution Reaction (HER), etc. They are sluggish kinetic reactions and require catalysts to depress the overpotential [6,7]. Precious metal-based catalysts, especially Pt-based catalysts, have high activity in electrochemical reactions [8]. Nevertheless, the poor stability caused by sintering, agglomeration, and poisoning and the high cost of precious metal catalysts are serious obstacles to their large-scale application [9,10]. Many efforts have been made to overcome such problems. Pt-based nanostructured catalysts have a high specific surface area, which can increase accessible atoms on the surface and, thus, improve Pt utilization [11]. Moreover, research shows that the ORR activity of the Pt(111) crystal plane is ten times that of Pt(100) and Pt(110) [12]. Therefore, through the control of Pt particle size and crystal plane exposure, the activity of Pt-based catalysts can be improved [13,14,15]. These physical, structural adjustments have improved the properties of Pt-based catalysts, but the thermodynamic instability caused by the high surface energy of nanoparticles makes them easy to aggregate. Furthermore, the inherently high oxygen adsorption energy of Pt makes the adsorption of oxygen on the platinum surface very strong [16], which is undesirable for an ideal catalyst [17]. Alloying Pt with transition metals can significantly change the binding energy of oxygen to the Pt surface [18]. However, the catalytic stability of alloy catalysts is still a challenge due to the poisoning caused by the dissolution of non-precious metals [19]. In addition, we have to admit that even the most ingenious design cannot change the scarcity and high cost of precious metals; for large-scale applications, we should minimize or even avoid the use of precious metals.

Non-precious metals based on transition metals have abundant reserves and low cost, so the development of non-precious metal or metal-free electrocatalysts is an important way to promote electrochemical energy to large-scale applications [20,21,22,23]. Transition metal compounds have attracted the attention of researchers as Pt-free electrocatalysts; however, the acidic medium in electrochemical reactions can seriously affect the properties of the compounds, causing them to be dissolved or corroded [24,25]. Transition metal-carbon composites have improved stability. Therefore, carbon-based non-previous metal catalysts are considered to be a promising alternative to precious metal catalysts due to their low cost, high activity, and durability [26,27,28,29]. The porous structure of carbon-based catalysts can increase the conductivity and dispersion of active species and contribute to the transfer of mass and electrons [30]. Porous carbon-based catalysts have gradually become an important electrocatalyst due to their good electrical conductivity, stability, large specific surface area, high porosity, and adjustable structure, and they have been widely studied for various electrocatalytic reactions [31,32,33]. A summary based on the extensive research on carbon-based non-previous metal catalysts is meaningful. Moreover, it might be logical to explain the synthesis of porous carbon materials in the beginning. In this review, the first part summarizes the synthesis of porous carbon materials which used as substrates for catalysts. The synthesis of porous carbon-based non-previous metal catalysts in recent years and their applications in electrochemistry are reviewed in the following sections, mainly including metal-free carbon-based catalysts, non-previous metal monatomic carbon-based catalysts, and non-precious metal nanoparticle carbon-based catalysts. Finally, we discuss the current problems and possible development directions of porous carbon-based non-previous metal catalysts. We hope that this systematic and organized review will give readers a quick overview of porous carbon-based non-previous metal catalysts and provide insights into their future development directions.

## 2. Preparation of Porous Carbon Materials

Carbon materials are excellent electrocatalyst supports due to their highly designable structures, suitable conductivity, and stability [34]. In recent years, the active sites in carbon-based electrocatalysts have been deeply studied through experiments and theoretical simulations, including their type, structure, and distribution. These achievements provide the impetus for reasonable selection and design of carbon matrix. The high specific surface area of porous carbon materials is conducive to the uniform dispersion of functional sites, and porous structures can effectively shorten the transport path of the reactant substances and reduce the transport impedance while also providing a larger contact area for the reactants to participate in the reaction fully [35]. In addition, porous carbon matrices can provide structural confinement, enabling the encapsulation of guest materials such as nanoparticles [36]. Therefore, the structural design of porous carbon substrates is one of the strategies to adjust the structure of electrocatalysts and improve their electrocatalytic performance. Taking the most common metal-nitrogen-carbon catalyst as an example, the electrocatalytic active sites include metal–Nx sites, heteroatom-doped carbon, and carbon-coated nanoparticles. In this regard, the large specific surface area and suitable pore structure of the carbon matrix are very important [37]. Thus, carbon material preparation technology plays an important role in promoting the development of electrocatalysts [38].

Therefore, we believe that it is necessary to review the synthesis methods of porous carbon materials, which are catalyst carriers, which will provide inspiration for adjusting the performance of carbon-based electrocatalysts.

### 2.1. Template Method

#### 2.1.1. Hard Template Method

**Figure 1:**
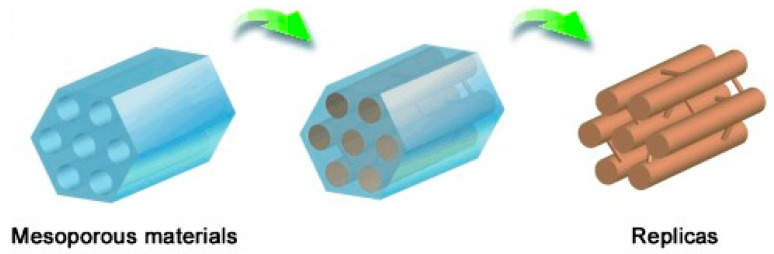
Schematic diagram of hard template synthesis process [40].

The hard template method is one of the common methods for the preparation of porous carbon, which can obtain a high specific surface area and ordered pore structure. It was first proposed by Ryoo et al. in 1999 and applied to the synthesis of mesoporous carbon materials [39]. The general steps for the preparation of porous carbon by hard template method are as follows: (i) select an initial template with the desired porous structure, (ii) mix the carbon precursor and the template under the structure guidance of the hard template, the carbon precursor enters the pores and solidifies, (iii) carbonize the mixture and remove the template to obtain the carbon material with desired porous structure. The hard template’s pore structure is reversed in the final porous carbon. The schematic diagram (Figure 1) clearly shows the synthesis process. Commonly used hard templates mainly include zeolites, mesoporous silica, and metal oxides. The porous carbon materials synthesized with different templates have different pore structures.

**Figure 2:**
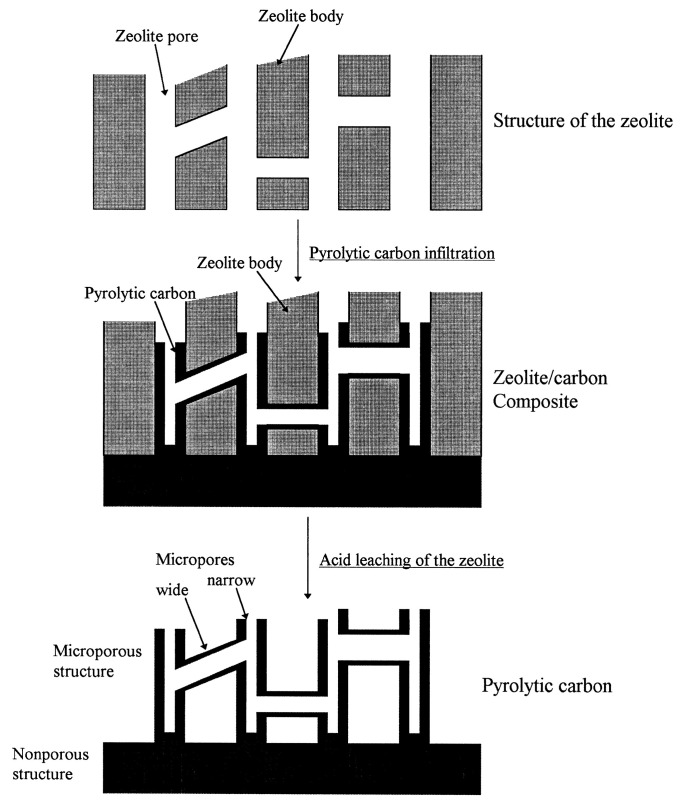
The synthesis process of microporous carbon materials with zeolite as a hard template [43].

**Figure 3:**
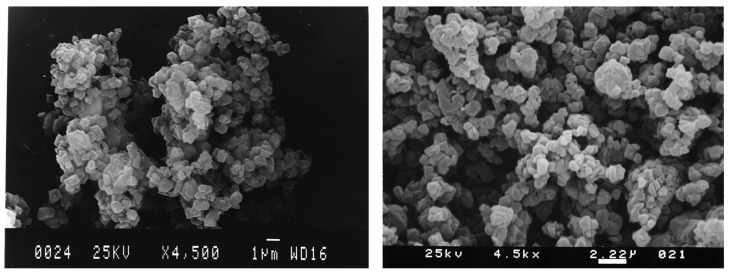
Scanning electron micrographs for zeolite (**left**) and carbon (**right**) [43].

Zeolite with an ordered sub-nanopore structure can be used as a template for ordered microporous carbon materials [41,42]. Rodriguez et al. [43] adopted zeolite Y as a template to prepare a microporous carbon. Figure 2 shows the synthesis process of zeolite-guided microporous carbon materials. The carbon precursor propylene was introduced into the micropores of zeolites by chemical vapor infiltration. Carbonization, followed by the removal of zeolite, yields microporous carbon. The resulting microporous carbon material has a high specific surface area of over 1300 m^2^ g^−1^. The SEM diagram (Figure 3) reveals that the morphology of the synthesized carbon material is similar to that of the original zeolite template, which indicates the template effect of zeolite.

Nueangnoraj et al. [44] used ordered zeolite as a template and introduced different quinone materials into the templating agent by simple impregnation to obtain microporous-rich carbon materials. The microporous carbon materials prepared from zeolite as a template have an energy density of up to 30.6 Wh kg^−1^ when used as capacitor electrode materials because of their uniform pore structure of 1.2 nm, which allows better storage of quinone materials. Lu et al. [45] used β-zeolite as a templating agent and ethylene as a carbon source and carbonized it at low temperatures to prepare nanoporous carbon materials with high specific surface area and abundant microporous structures, which are suitable for energy gas storage, adsorption, and separation of small molecules.

Due to the uniform pore walls with a thickness of less than 1 nm, zeolites are powerful templates for the synthesis of microporous carbons. However, the small pores of zeolites lead to the thin walls of the synthesized carbon materials, and a certain amount of mesopores may be generated due to the partial collapse of the carbon framework during the removal of zeolites. Studies have shown that the inheritance of zeolite structure by carbon materials depends on the type of zeolite. When zeolite with large micropores (>0.6 nm) is used as a template, microporous carbon materials with high structural regularity can be obtained [46]. Nevertheless, the microporous structure of zeolite template carbon materials is not favorable for the diffusion of reactants.

**Figure 4:**
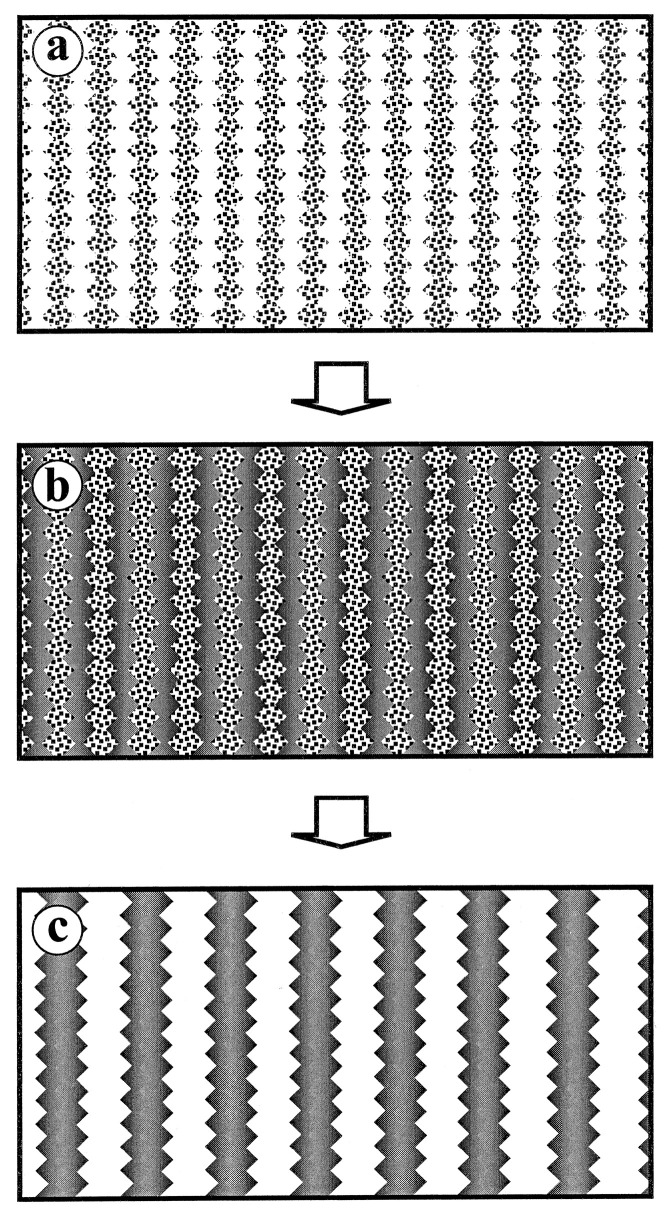
The synthesis process of mesoporous carbon materials with mesoporous silica as a hard template (**a**) The mesoporous silica MCM-48, (**b**) MCM-48 after completing carbonization, and (**c**) carbon obtained by removing the silica wall [53].

Mesoporous silica has uniform and interconnected pores and is used as a template for the synthesis of mesoporous carbon materials. Commonly used mesoporous silica templates include SBA-15 [47,48,49] and KIT-6 [50,51]. The earliest carbon material obtained using it as a template dates back to 1998. Zakhidov et al. [52] used porous silica opal crystals as a template and phenolic resin and propylene as a carbon source to prepare the inverse opal material. In 1999, Ryoo et al. [53] used MCM-48 as a template, sucrose as a precursor, and sulfuric acid as a catalyst to prepare carbon material CMK-1 with uniform pore size and specific surface area as high as 1800 m^2^ g^−1^, which can be used as an adsorbent for gas and liquid pollutants. Figure 4 shows the template strategy for the synthesis of ordered mesoporous carbon materials using mesoporous silica templates. Similarly, CMK series porous carbon materials such as CMK-3, CMK-5, CMK-4, and CMK-2 have regular and ordered pore structures, ultra-high specific surface area, and large pore volume. In addition, the researchers also prepared mesoporous carbon nanospheres [54] and tubular mesoporous carbon [55] using FDU-12 and FDU-5 as hard templates, respectively.

Etching silicon templates often requires the use of strong alkalis or highly toxic hydrofluoric acid, which has problems with equipment corrosion and safety. Metal oxides such as alumina, magnesium oxide [56] and zinc oxide [57] can be dissolved in dilute acids and can, therefore, be used instead of silicon materials in the fabrication of porous carbon materials. Using MgO as a template and polyethylene terephthalate as a carbon source, Przepiorski et al. [58] prepared mesoporous carbon materials with different yields by adjusting the amount of polyethylene terephthalate and MgO. Wang et al. [59] used alumina (AAO) nanorods as a template and melamine as a carbon and nitrogen source to condense melamine within the nanopore channel and generated g-C_3_N_4_ nanorods (CNR) by the nano-limiting effect of AAO. The nano-limiting effect of the AAO template enhanced the orientation and crystallinity of CNR, which resulted in CNR exhibiting a more desirable C/N ratio.

**Figure 5:**
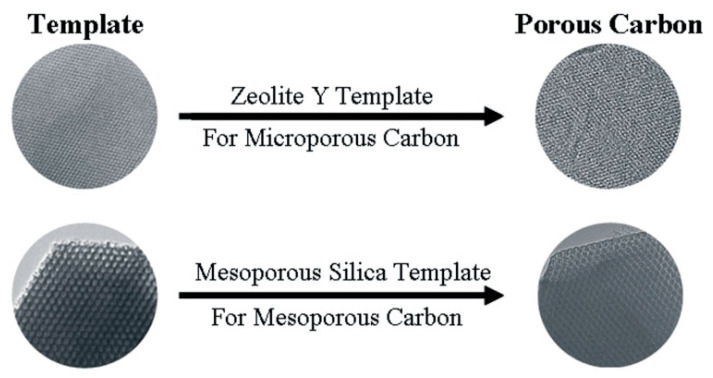
Conceptual schematic diagram of zeolite and mesoporous silica as hard templates for the synthesis of porous carbon [60].

The hard template method is essentially a replication process, and the structure of the resulting carbon material depends on the pore structure of the template (Figure 5). Table 1 summarizes the template, BET-specific surface area, pore size, and carbonization conditions of various carbon materials.

When choosing a suitable hard template, we must consider two factors: (i) whether the template can maintain its pore structure during the carbon precursor filling process and (ii) whether the templating agent can be conveniently deposited without destroying the carbon material. Although the carbon materials prepared by the hard template method have the advantages of adjustable pore size and orderly structure, the preparation method is tedious and requires strong acid or strong base to etch the templating agent, which has the defects of high cost and low efficiency, limiting its large-scale industrial application.

#### 2.1.2. Soft Template Method

The soft template method is a method based on organic self-assembly at the molecular level. Soft templates (such as organic molecules or supramolecules) self-assemble with liquid carbon precursors through hydrogen bonding and hydrophilic/hydrophobic or electrostatic interactions to form mesoscopic structures, which are pyrolyzed and carbonized to obtain porous carbon materials [61]. The soft template method has stringent specifications for carbon precursors and templates, which include the following three conditions: (i) the templating agent must provide a sufficient driving force to form micelles; (ii) the precursors can be polymerized or cross-linked to stabilize the pore structure skeleton; (iii) the precursors should be more thermally stable than the templating agent to avoid collapse of the carbon structure during carbonization [62]. Compared with the hard template method, the soft template method can hardly synthesize ordered porous carbon, but this method does not require the template etching step and has mild reaction conditions, so it is widely used. Commonly used templating agents for soft templates include ionic surfactants and block copolymers.

Ionic surfactants rely on electrostatic interaction to realize self-assembly with liquid carbon precursors. Initially, cationic quaternary ammonium salts were used as soft templates. Later, Moriguchi et al. [63] used phenolic resin as a surfactant. Positively charged ionic surfactant CTAB reacts with negatively charged phenolic resin to form a mesoscopic polymer. Layered, hexagonal, and disordered polymers were formed by systematically adjusting the ratio of phenol to CTAB (from 1:1 to 6:1). Subsequently, Zheng et al. [64] synthesized microporous carbon Pt-s-C using resorcinol-formaldehyde resin, platinum-based complexes, and surfactant CTAB as raw materials, and Pt-s-C catalyzed the CO reaction at 175 °C with a conversion of up to 99%. However, the weak electrostatic interaction force of ionic surfactants usually leads to the collapse of the carbon structure [65].

**Figure 6:**
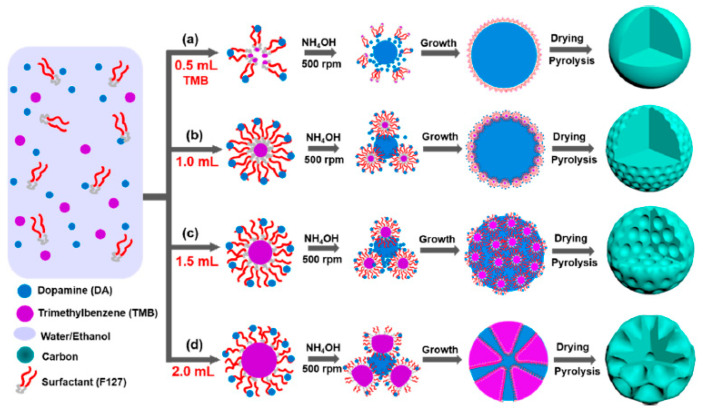
Flowchart of the synthesis of N-doped carbon nanospheres with variable pore size (**a**) 0.5mL TMB; (**b**) 1.0mL TMB; (**c**) 1.5mL TMB; (**d**) 2.0mL TMB [68].

Block copolymers are considered efficient soft templating agents since they can self-assemble with liquid precursors to obtain aggregate molecular structures. Unlike ionic surfactants, block copolymers depend on hydrogen bonding to achieve self-assembly with liquid precursors. Amphiphilic triblock copolymers are the most typically utilized soft-templating agents [66,67]. Meng et al. [68] used Pluronic F127 as a soft template, dopamine as a carbon source, and 1, 3, 5-trimethylbenzene (TMB) as a modulator to synthesize a series of N-doped carbon nanospheres with tunable pore size in a mixed solvent system of water and ethanol (the preparation flowchart is shown in Figure 6). The TMB has a very important influence on the regulation of pore size, and the regulation of its amount can effectively regulate the assembly of micelles. N-doped carbon nanospheres have various morphologies, such as smooth carbon spheres, golf ball-shaped carbon spheres, and multi-cavity carbon spheres. This simple nanoemulsion synthesis approach opens up a new avenue for the fabrication of novel mesoporous materials.

The pore structure of carbon synthesized by a hard template represents the inverse structure of a template, so the pore wall thickness of a hard template determines the pore size of carbon material. However, in the soft template method, the pore size can be adjusted by rationally selecting the template polymer. In addition, larger pores can be produced by adding an expansion agent.

The template method can prepare porous carbon-based catalysts with ordered and continuous channels, but the preparation process using the hard template method is time-consuming, complicated, and expensive, while the soft template method has high requirements for the templating agent.

#### 2.1.3. Activation Method

The activation method is a common and well-established method for preparing porous carbons. Compared with the template method, the activation method does not need a template, so it does not need a template processing step. The morphology of carbon materials prepared by the activation method is more diversified. In addition, the activation method has a low raw material cost, so it is widely used to prepare porous carbon materials.

**Figure 7:**
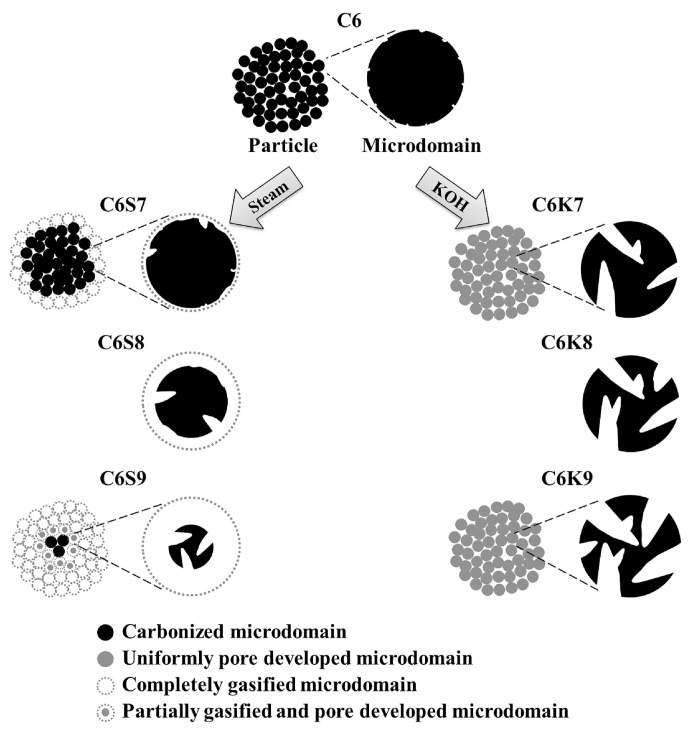
Mechanism model of physical activation and chemical activation [70].

Activation methods are often divided into physical activation and chemical activation methods (Figure 7). The physical activation method is that the gaseous medium (water vapor, carbon dioxide, and air) gasifies part of the carbon atoms in the carbon material at high temperatures to obtain a porous material. The pores of the porous carbon prepared by this method are mostly in the amorphous carbon region, and the pore structure is generally not adjustable. Lua et al. [69] used oil palm stone as the carbon source to prepare porous carbon materials by the CO_2_ physical activation method. The results revealed that the particle size and heating rate of the carbon precursors had no substantial influence on the specific surface of carbon, while the CO_2_ flow rate, activation time, and activation temperature can influent the specific surface significantly. Under the optimal activation conditions (850 °C, 2 h), the specific surfaces and microporous volumes of the catalysts were 1410 and 942 m^2^ g^−1^, respectively.

**Figure 8:**
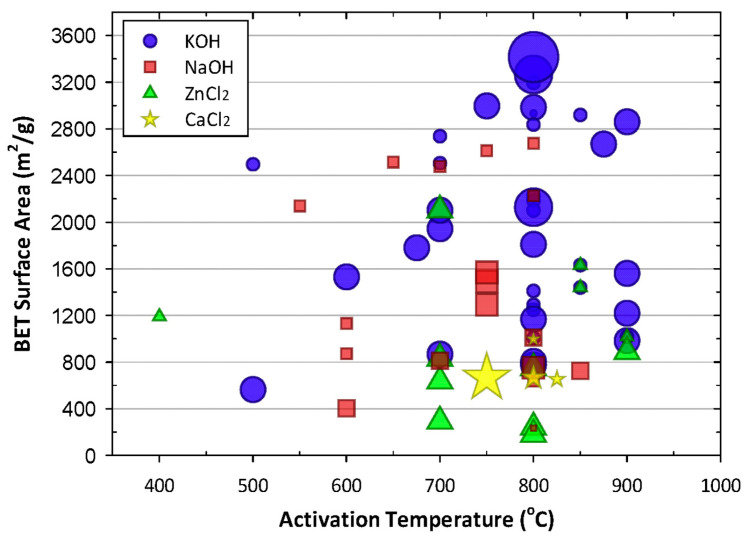
BET surface area of porous carbons through KOH, NaOH, ZnCl_2_, and CaCl_2_ agents at various temperatures [75].

The chemical activation method uses chemical reagents as activators to carbonize at a certain temperature, and the carbonization process and the activation process are carried out simultaneously. The commonly used activators include acids (phosphoric acid), strong bases (potassium hydroxide), and chloride salts (zinc chloride) [71,72,73]. Among the strong base activators, potassium hydroxide is the most effective activator, which reacts with carbon at high temperatures and can etch carbon materials and, thus, obtain microporous carbon materials with a high specific surface. However, the amount of strong alkali activator added is generally several times that of carbon material, and the material needs to be acid and water-washed after high-temperature pyrolysis, which increases the processing steps and may cause corrosion of equipment. In contrast, the additional amount of zinc chloride as an activator for the generation of porous carbon materials is lower than that of a strong alkali catalyst. Yu et al. [74] used zinc chloride to activate phenol-formaldehyde polymers, and the activation process and carbonization process were carried out simultaneously, and the phenol-formaldehyde polymer precursors were finally transformed into foam-like carbon aerogels, and the obtained carbon aerogels had low density (25 mg cm^−3^) and high specific surface area (1340 m^2^ g^−1^), as well as large micro- and mesopore volumes (0.75 cm^3^ g^−1^). Figure 8 shows the specific surface area of porous carbon obtained by the activation of various reagents at different temperatures. Compared with the template method, the activation method is straightforward to prepare, and the prepared carbon materials have a high specific surface, but the pore size is small and mostly microporous structure, which limits the application of some large guest molecules.

#### 2.1.4. Direct Pyrolysis Method

Direct pyrolysis is a method to burn carbon precursors directly at high temperatures. The fabrication process of this method is straightforward, and the pore structure of the obtained carbon materials is related to the precursors, so the selection of carbon precursors is very critical. Commonly used carbon precursors include metal organic skeletons, biomass, polymers, small molecule compounds containing carbon atoms, etc. Biomass, as a renewable energy source, has the advantages of plentiful reserves and economic and environmental protection, so it is often used in the fabrication7 of porous carbon materials [76,77,78]. For instance, Cai et al. [78] prepared nitrogen-doped porous carbon (NAC) by direct pyrolysis using agricultural waste corn cobs as the carbon source to obtain microporous-based activated carbon materials with a specific surface of up to 2859 m^2^ g^−1^ and a specific capacity of up to 129 mA h g^−1^ (185 F g^−1^) at a current density of 0.4 A g^−1^, along with excellent multiplicative performance and cycling stability.

**Figure 9:**
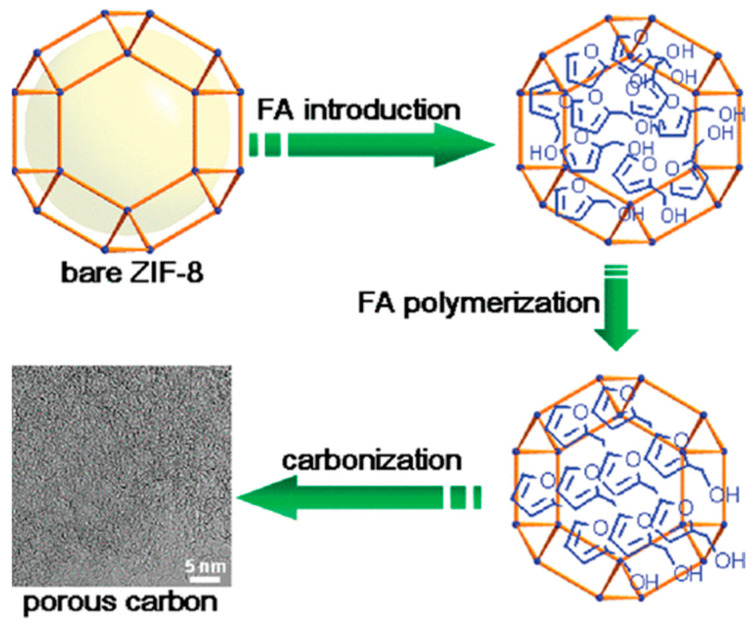
Schematic diagram of porous carbon preparation process using MOF as hard template [79].

Metal organic backbone (MOF) is a novel crystalline porous material that can act as both a carbon precursor and a template by benefiting from the high carbon content of organic ligands. Jiang et al. [79] prepared porous carbon materials with a high specific surface area (3405 m^2^ g^−1^) using ZIF-8 as a template. A schematic diagram of the synthesis process is shown in Figure 9. Due to the small pore size of ZIF-8, the loading of carbon precursors is limited, but this work reveals the potential of MOF-derived carbon materials. Chen et al. [80] prepared catalysts with interconnected pore structures with an average pore size of 2.5 nm and up to 17.82% nitrogen doping by pyrolyzing carbon nanotube-linked ZIF-8 precursors under a nitrogen atmosphere. The specific capacitance was up to 426 F g^−1^ at a current density of 1 A g^−1^ in 1 M H_2_SO_4_ solution, which showed outstanding stability in the 10,000 cycles test. Huang et al. [81] used Zn-MOF-74 as the carbon source, melamine as the nitrogen source, mixed MOF and organic small molecules, and calcined directly to obtain nitrogen-doped porous carbon materials (NPC). By changing the calcination temperature and calcination time, the nitrogen doping types (pyridine nitrogen and graphite nitrogen) in NPC can be effectively adjusted. The rich pore structure of carbon materials is conducive to the full exposure of active components to the electrolyte and to the efficient reduction of CO_2_ for CO preparation. The direct pyrolysis method uses the release of a large amount of pyrolysis gas during the pyrolysis process to increase the porous structure of carbon materials and improve the specific surface area of porous carbon materials. It is the most commonly utilized in the preparation of porous carbon materials.

Direct pyrolysis is beneficial to obtain catalysts with high electrical conductivity and good corrosion resistance, but the aggregation of metals and the inevitable destruction of pore structure may lead to a decrease in the active center and specific surface area. More effective synthesis strategies are still needed to avoid these shortcomings.

The development of synthetic methods on carbon substrates can facilitate the structural optimization of electrocatalysts. The large specific surface area of the porous carbon matrix is beneficial to the dispersion of catalytic sites, while the porosity is very important for mass transport and electron exchange. Specifically, the micropore structure can increase the specific surface area, thus, exposing more active sites. Micropores are beneficial to provide a large number of active catalytic centers but are not conducive to mass transfer, so the utilization of catalytic sites in micropores is usually low. In the rational design of carbon matrix pore structure, we should comprehensively consider the active role of macropores in mass transfer and the large number of active centers brought by micropores. The optimization of electrocatalytic performance can be achieved by designing the microstructure of carbon substrates by rationally combining the characteristics of different pore sizes.

## 3. Research Progress of Porous Carbon-Based Non-Precious Metal Electrocatalysts

### 3.1. Metal-Free Carbon-Based Catalysts

**Figure 10:**
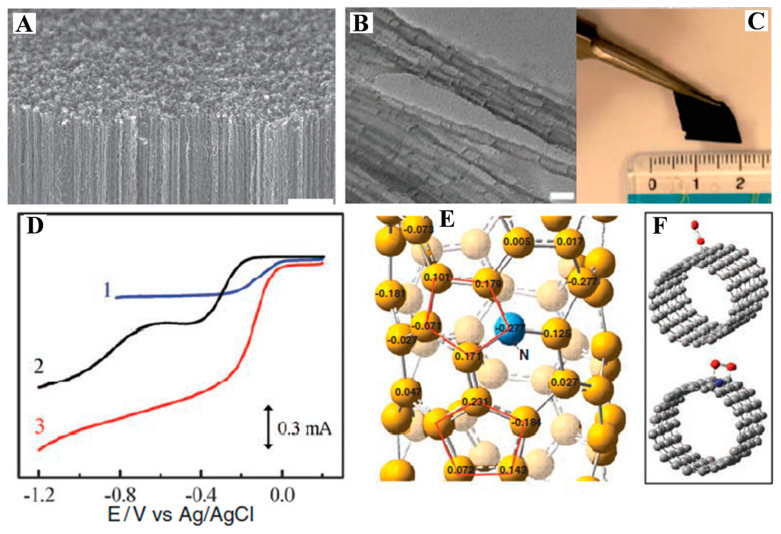
(**A**) SEM images of VA-NCNTs, (**B**) TEM images of VA-NCNTs, (**C**) photos of VA-NCNTs, (**D**) ORR linear sweep curve of Pt-C/GC (curve 1), VA-CCNT/GC (curve 2) and VA-NCNT (curve 3), (**E**) calculated charge distribution of VA-NCNTs, and (**F**) the possible adsorption mode of oxygen molecules on VA-NCNTs [87].

Carbon-based materials have attracted more and more attention due to their unique electronic properties, tunable pore structure, high specific surface area, and excellent electrical conductivity [82]. It is well known that metal catalysts are easy to be corroded in an acidic environment, thus, affecting their catalytic stability. Carbon-based metal-free catalysts may be the hope of high-stability electrocatalysts in an acidic environment [83]. One of the approaches to improve the electrocatalytic activity of carbon substrates is to dope them with heteroatoms [84]. Among the different heteroatoms, N-doping makes the most promising model [85,86]. In 2009, Dai et al. [87] reported an N-doped carbon nanotube vertical array catalyst (VA-CNTs) in Science, which has a current density of up to 4.1 mA cm^−2^ at −0.22 V. Figure 10 shows the morphology characterization, electrochemical tests and theoretical calculation results of the catalyst. The catalyst has better activity than Pt/C under alkaline conditions and is resistant to both CO and methanol poisoning. The calculation results show that the doping of electron acceptor nitrogen makes the atoms on the carbon planes of the conjugated nanotubes exhibit relatively high positive charge, which increases the charge density of adjacent carbon atoms and facilitates oxygen adsorption, and also weakens the O-O bond and reduces the catalyst’s decisive step energy barrier, thus, enhancing the ORR performance.

Since then, metal-free electrocatalysts represented by N–C have been extensively studied. Nitrogen-doped carbon materials have been demonstrated to exhibit outstanding ORR activity (Table 2). With five valence electrons, N can be considered an n-type carbon dopant, and the incorporation of N can change the electronic structure of C and serve as active sites to facilitate the electrocatalytic process [88].

Nitrogen doping is an important means to modify graphene, which has a wide application prospect because of its low cost and high controllability. N atom and C atom are close neighbors in the periodic table, and their atomic radius and electronegativity are similar, so the doping of the N atom into graphene lattice will not cause a big change in graphene lattice, and it is easy to achieve doping. Nitrogen-doped graphene can be obtained by direct synthesis and post-treatment. Most of the nitrogen doping by post-treatment will only lead to the heterogeneous doping of graphene. Although doping by direct synthesis can produce homogeneous doping in principle, the results reported so far cannot fully prove this point. Specifically, the direct synthesis methods include the CVD method [98], the segregation growth method [99], the solvothermal method [100], and the arc discharge method [101]. The post-treatment methods include heat treatment [102], plasma treatment [103] and N_2_H_4_ treatment [104].

In 2016, Nakamura et al. [105] designed graphite model catalysts (highly oriented pyrolytic graphite HOPG) that could be prepared in four types: (i) pyridine N-doped HOPG (pyri-HOPG); (ii) graphite N-doped HOPG (grap-HOPG), (iii) defective HOPG; (iv) HOPG for probing the impact of nitrogen doping type on the activity of carbon materials. The results show that the pyridine nitrogen-doped carbon material has unsurpassed ORR activity under acidic conditions, and the doping of pyridine nitrogen makes the catalyst basic, and the basic sites are mostly found on the carbon atoms adjacent to the pyridine nitrogen, so the ORR active sites in the N-doped carbon material are the carbon atoms adjacent to the pyridine nitrogen with Lewis basicity.

Immediately after, Yang et al. [106] prepared metal-free 3D reticulated graphene nanoribbons (N-GRW) and used them in ORR and OER reactions to further investigate the effects of different nitrogen doping types of N-GRW on ORR and OER activities, and the experimental results showed that electron-absorbing pyridine N is the active site of OER while electron-donating graphite N is the active center of ORR. The peculiar 3D nanoribbon structure facilitates the transfer and transport of electrons and electrolytes. As a cathode catalyst, it was employed for zinc-air batteries with an open-circuit voltage of 1.46 V, a specific capacity of 873 mAh g^−1^, and a maximum power density of 65 mW cm^−2^. Therefore, N-GRW can be utilized as a low-cost and high-efficiency air electrode cathode material having a great deal of potential for application in rechargeable metal-air batteries.

Zheng et al. [107] proved that nitrogen-doped graphene-supported graphite phase carbon nitride (g-C_3_N_4_@NG) could be used as a metal-free catalyst for hydrogen evolution. The results of electrochemical tests and thermodynamic calculations show that the excellent electrochemical energy lies in its hybrid structure, and the graphite phase carbon nitride can provide highly active hydrogen adsorption sites, which promotes the proton-electron transfer process and achieves a faster HER reaction kinetics.

**Figure 11:**
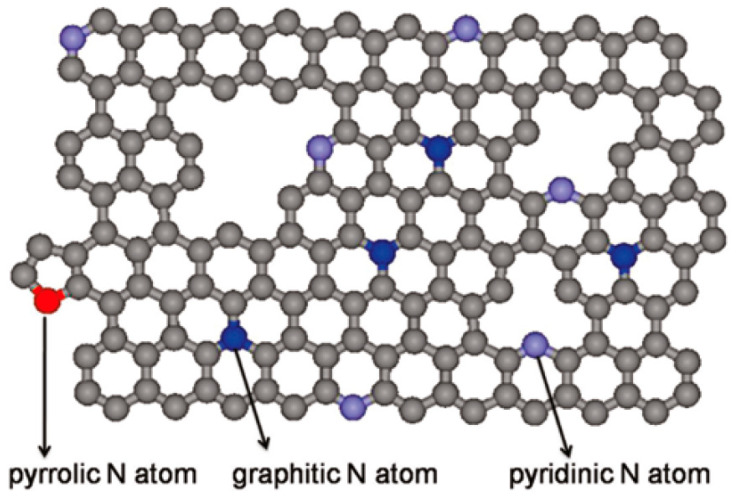
Schematic diagram of different nitrogen doping types in carbon materials [108].

It can be seen that the oxygen reduction catalytic activity of nitrogen-doped carbon catalysts is closely connected to the amount and the form of nitrogen doping. As illustrated in Figure 11 [108], the nitrogen-doped into the carbon matrix can be mainly classified as pyridine nitrogen, pyrrole nitrogen and graphite nitrogen, and a small portion of pyridine oxide nitrogen. Pyridine nitrogen is in a six-membered ring and is attached to two carbon atoms; in this structure, pyridine nitrogen provides one electron to form a conjugated π-bond and also a pair of lone electrons remaining (easy to form coordination bonds with transition metals); pyrrole nitrogen is in a five-membered ring with four carbon atoms, pyrrole nitrogen is attached to a hydrogen atom, and the remaining two electrons in the p-orbitals form a conjugated π-bond; graphite nitrogen is a carbon atom in the substitution plane attached to three surrounding carbon atoms. Different types of doped nitrogen have different roles in the oxygen reduction reaction process, and it is generally thought that pyridine nitrogen or pyrrole nitrogen is the active site of the reaction, while pyridine oxide nitrogen has no role. However, there is still some research that considers graphitic nitrogen as the main active source of ORR. So far, there is still controversy about the active site.

N-doping can shift the Fermi surface of graphene, thus, opening the band gap while realizing n-type doping. In addition, N-doped graphene may have some specific pore structures, which can increase the surface active adsorption site density of graphene and enhance the adsorption of metal particles or gases on the surface of graphene. This characteristic makes N-doped graphene have superior electrochemical performance and can be developed into high-performance electrode materials.

The regulation of the carbon matrix is another important way to improve the catalytic activity. Jiang et al. [109] achieved the preparation of nitrogen-doped ultrathin carbon nanosheets (NCN-1000-5) by pyrolysis of a mixture of citric acid and ammonium chloride (mass ratio of 1:1) using a spontaneous gas production method. The carbon nanosheets obtained under 1000 °C pyrolysis conditions have ultrathin sheet structure, ultra-high specific surface area (1793 m^2^ g^−1^), and abundant defect structure, and exhibit low overpotential and high cycling stability to ORR, OER, and HER. Assembled as a catalyst for rechargeable Zn-Air batteries, NCN-1000-5 exhibits high energy density (806 Wh kg^−1^), low charge/discharge voltage gap (0.77 V), and excellent cycling stability at 10 mA cm^−2^.

Iodine-doped graphene is another important metal-free carbon-based electrocatalyst. Since the atomic radius of iodine is larger than that of carbon and iodine doping involves complicated surface charge transfer, iodine doping is more complex than nitrogen doping. Density functional theory shows that as p-type surface dopants, I_3_ and I_5_ can move the Fermi level below the Dirac point to 0.46 eV and 0.57 eV [110]. Iodine-doped graphene oxide has attracted much attention as a fuel cell (FC) electrocatalyst with high activity for oxygen reduction (ORR) [111,112,113].

In 2011, Kalita et al. [114] used the CVD method to grow graphene thin films and realized iodine doping in them. In this technique, iodine is mixed with carbon precursors and evaporated at the same time, which overcomes the difficulty of in-situ iodine doping in graphene films in the traditional chemical vapor deposition process using gas sources. Iodine exists in the form of triiodide and pentaiodide, and iodine atoms can interact with carbon atoms through charge transfer. This work shows that atomic iodine can be located on the surface or embedded in the form of polyiodide without interfering with the hexagonal lattice structure of graphene films and can form iodine-carbon interaction with carbon atoms, which inspires the study of iodine-doped graphene.

In 2015, Zhan et al. [115] tested the electrochemical performance of I-doped graphene as the anode material of lithium-ion batteries for the first time. Iodine-doped graphene is synthesized by heat treatment of a mixture of graphite oxide and iodine. Compared with undoped graphene, I-doped graphene shows high reversible capacity, long-term cycle performance, and excellent rate performance at very high current density. After doping, the d spacing of graphene increases, and the structural defects and the positive charge density are introduced on the surface of graphene, which leads to richer lithium-ion storage, rapid lithium-ion diffusion, and electron transport. This work reveals the potential of I-doped graphene for lithium-ion batteries.

Adriana et al. [116] reported the catalytic performance of iodine-doped graphene in ORR. I-doped graphene was prepared by nucleophilic substitution of graphene oxide reduced by HI, and it shows a high surface area, mesopore, and vacancy. Structural characteristics and their synergistic effects can not only improve ion and electron transport but also limit ohmic resistance. Therefore, I-doped graphene shows excellent electrochemical performance and long-term stability, which indicates that I-doped graphene has great potential to be a high-efficiency electrode material.

Multicomponent heteroatom doping (like B, S, and P) is an effective method to regulate the catalytic activity and selectivity of carbon-based catalysts [117,118,119]. Co-doping electron-donating atoms (such as S or P) with electron-accepting atoms (such as N) can prepare bifunctional catalysts [120,121]. More active centers are introduced by changing the electronegativity, charge distribution, and electron transfer behavior of the carbon matrix [121]. Meanwhile, heteroatom doping also introduces a large number of structural defects to the carbon matrix, which improves catalyst activity.

Hou et al. [117] prepared N, B co-doped graphitic carbon nanocages with unique defect structures and confirmed theoretically and experimentally the important value of N, B co-doped graphitic carbon materials in the multi-functional catalytic reactions of ORR, OER, and HER, that is, NB-CN’s unique semi-open nano-cage structure and defect-rich ordered graphite structure can increase the effect of charge transfer and provide more active sites to enhance the catalytic activity.

**Figure 12:**
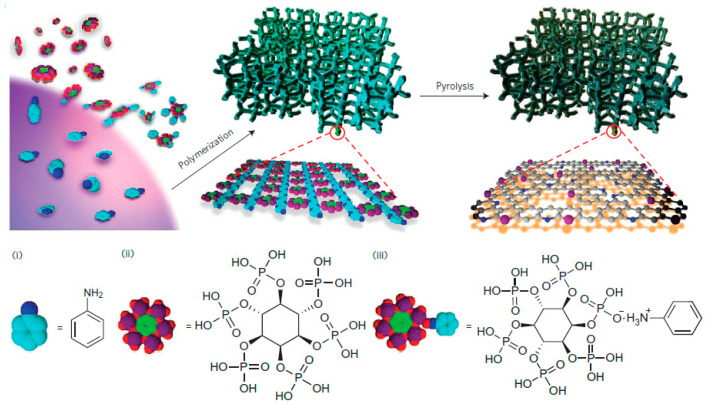
Schematic illustration of the preparation process for the NPMC Foams An aniline (i)–phytic acid (ii) complex (iii) is formed [118].

Dai et al. [118] reported a template-free method for the preparation of three-dimensional nitrogen-phosphorus co-doped mesoporous materials. In this method, polyaniline and phytic acid are first formed into airgel and then carbonized at high temperatures to obtain N, P double-doped carbon materials. Figure 12 depicts the process of this template-free method. The air battery assembled with this carbon material has high open circuit voltage and energy density. The first-principle simulation calculation results show that the N, P co-doped porous carbon network has dual functions and efficient ORR and OER activities.

Yang et al. [119] rationally designed a sort of catalysts with oxygen reduction activity at full pH, namely N, S co-doped porous carbon nanosheets (NSPCS), by modulating the type and content of N and S doping. Electrochemical experiments showed that NSPCS has a high ORR half-wave potential (E_1/2_ ≈ 0.75 V) in acidic media. Further results of structural characterization of NSPCS show that there are abundant defect structures on the surface, and the N and S co-doping optimizes the structure of the catalyst with excellent catalytic performance in both oxygen reduction reactions and supercapacitor applications. In addition, NSPCS exhibits outstanding methanol resistance and also good stability, further demonstrating that a carbon-free metal catalyst is a favorable option for replacing commercial Pt-based catalysts in practical fuel cells.

Carbon-based metal-free catalysts have better acid resistance than transition metals, and the catalytic activity of many kinds of carbon-based metal-free catalysts is now comparable to that of metal-based catalysts. At present, the understanding of the active center in the carbon-based metal-free catalyst is still insufficient, which needs systematic and in-depth research. Nevertheless, it can be predicted that the development of carbon-based metal-free catalysts will be conducive to the commercial application of chemical energy and will be an important development direction of electrocatalysts in the future.

### 3.2. Non-Precious Metal Nanoparticle Carbon-Based Catalysts

During the preparation of catalysts by pyrolysis, transition metal-containing precursors can easily form metal nanoparticles, and heteroatoms will be incorporated into carbon materials to form defective carbons. Transition metal nanoparticles/carbon-doped composite electrocatalysts have been widely studied in recent years because of their high conductivity, high specific surface area, and corrosion resistance [122]. Nanoparticles/doped carbon composite catalysts can be divided into two types: one containing exposed nanoparticles and the other containing graphite carbon layer-wrapped nanoparticles [123]. Due to long-term contact with electrolyte solution, the exposed nanoparticles are very prone to oxidation and dissolution, resulting in poor activity and stability of the catalyst, while the coated carbon layer can protect the nanoparticles [61]. Some studies attribute the oxygen reduction activity to the synergistic effect of nanoparticles and outer-coated carbon. Through the calculation results, it is found that there is a phenomenon of electron transfer between nanoparticles and coated carbon layer, which promotes the oxygen reduction reaction. Therefore, the performance of the catalyst is mainly affected by two aspects. On the one hand, is the structure and composition of the carbon matrix; for example, the porosity of the carbon matrix affects mass transfer, electron transport, and exposure of active sites [62,63]. On the other hand, nanoparticles promote the catalytic oxygen reduction reaction by forming a synergistic effect with the active sites in the outer coated carbon layer or the surrounding carbon matrix. Therefore, the type, size, and distribution of nanoparticles are important factors affecting the electrocatalytic activity, as well as the pore structure and morphology of the carbon matrix.

#### 3.2.1. Iron Carbide Nanoparticle/Carbon Composite Electrocatalyst

**Figure 13:**
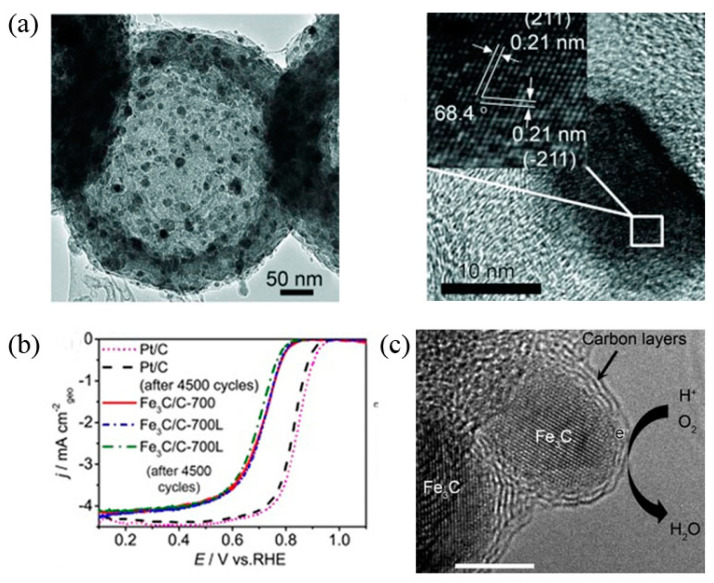
(**a**) Representative examples of carbon-encapsulated iron carbide particles promote ORR catalytic activity, (**b**) ORR polarization curves, and (**c**) the oxygen reduction process on the catalyst [124].

Carbon-encapsulated iron carbide particle catalysts are frequently employed in oxygen reduction reactions. Li et al. [124] published a significant study demonstrating the catalytic ORR contribution of such encapsulated structured iron carbide particles (Figure 13). The authors used a one-step high-pressure pyrolysis method to prepare catalysts with hollow spherical structures Fe_3_C/C, which had essentially negligible nitrogen and iron content on the surface, and the iron carbide nanoparticles were encapsulated by 4–9 layers of graphitic carbon layers. In alkaline electrolytes, the catalytic activity of Fe_3_C/C-800 for ORR is the same as that of commercial Pt/C catalysts. In acidic electrolytes, Fe_3_C/C-800 exhibited relatively high onset and half-wave potentials, about 100 mV lower than the Pt/C catalyst. Similar to the Pt/C catalyst, this catalyst also exhibited a low Tafel slope and a four-electron ORR reaction process. Moreover, the catalyst Fe_3_C/C-800 exhibited excellent stability in both acidic and basic media. The authors proposed that although the iron carbide nanoparticles do not come into direct interaction with the electrolyte and reactants, they still play an important role in the oxygen reduction reaction by activating the outer encapsulated graphitic carbon layer. Similar results, such as Fe_3_C@N-C-900 synthesized by Liu et al. [125] also exhibited efficient electrocatalytic activity and stability not inferior to that of Pt/C catalysts.

**Figure 14:**
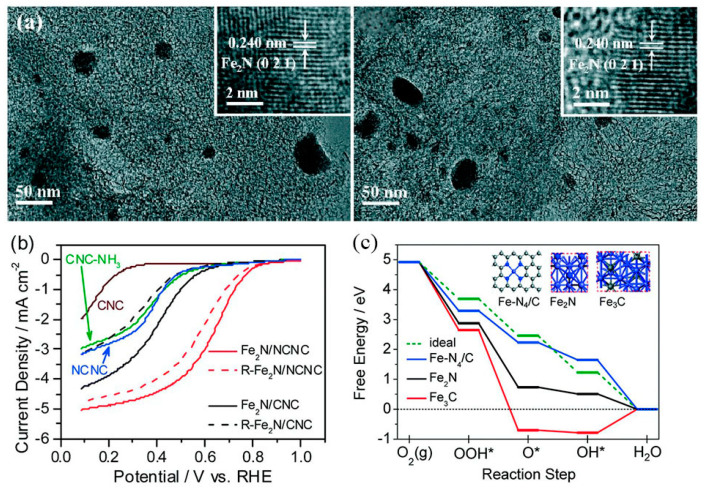
(**a**). TEM images of Fe_2_N nanoparticles loaded on Carbon nanocages (CNCs) and nitrogen-doped CNC (NCNC), (**b**) ORR performance of the Fe_2_N/NCNC and Fe_2_N/CNC catalysts, and (**c**) free energy diagrams of the ORR process [127].

The catalytic sites that confer high ORR activity are an interesting subject. Kramm et al. [126] significantly removed the inorganic metal species in the catalyst by secondary heat treatment and acid leaching and prepared a catalyst containing only FeN_4_ sites, which has a greatly improved ORR activity. Due to reducing the contribution of metal species to zero, the experimental results reveal that the Fe–NX/C moiety (x ≥ 4) is the active center for ORR. Sun et al. [127] designed the Fe_2_N and Fe_3_C-based catalysts with or without Fe-Nx (Figure 14). By systematically comparing the ORR activity, it was found that only the former had high ORR activity. The results show that similar to Fe/N/C catalysts, Fe–N-related species are the reason for the high ORR activity of iron-based catalysts. Density functional theory calculations also support that the ORR activity of Fe–N_4_/C is much higher than that of Fe_2_N and Fe_3_C. This study shows that the high activity of iron-based catalysts comes from the Fe–Nx/C part (x ≥ 4) rather than the Fe_2_N or Fe_3_C phase.

**Figure 15:**
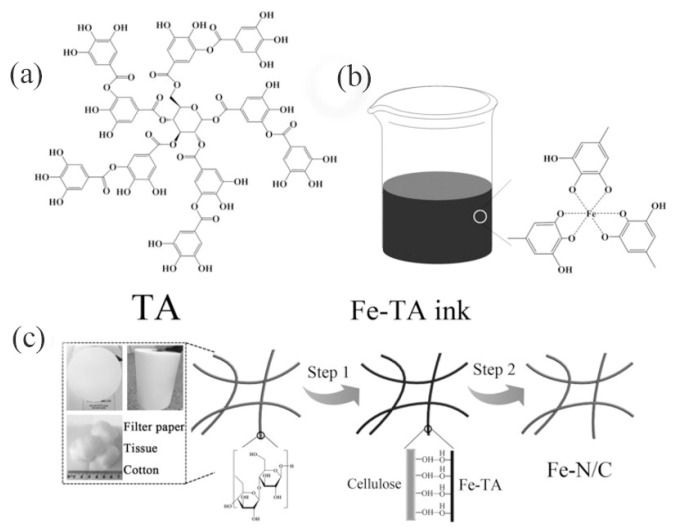
(**a**) The structural formula of tannic acid (TA); (**b**) the chemical structure of Fe–TA–framework ink and its schematic depiction in a beaker; (**c**) the fabrication process [132].

Heteroatom-doped carbon fibers containing iron carbide particles are commonly used electrocatalysts, which are mostly synthesized by the electrostatic spinning technique [128,129,130]. Ren et al. [128] used an electrostatic spinning technique to synthesize porous nitrogen-doped carbon nanofibers with core-shell structure encapsulating iron carbide. The onset and half-wave potentials of this catalyst exceeded those of commercial Pt/C catalysts in alkaline electrolytes, but the catalytic activity for oxygen reduction was not satisfactory in acidic media. Guo et al. [129] synthesized three-dimensional composite carbon nanowire iron carbide catalysts by introducing phosphorus elements into pyrolytic electrospun fibers (PVA/H_3_PO_4_/Fe(AC)_2_). The catalyst demonstrated improved ORR catalytic activity in acidic media, and the authors proposed that the outstanding catalytic activity was due to the abundant active sites (Fe_3_C@C, Fe–P, and P–C) and multilevel pore structure. Zhong et al. [130] introduced MIL-88B-NH_2_ in polyacrylonitrile fibers using the electrostatic spinning technique, and pyrolysis yielded catalysts with a large number of uniformly dispersed iron carbide particles and abundant pore structure; the catalyst also exhibited excellent oxygen reduction catalytic activity in acidic media. Hence, the plentiful active sites and multi-level pore structure are important factors in improving the catalyst activity. Moreover, nanofibrous catalysts can also be prepared by adding biomass carbon fibers [131,132]. For example, polypyrrole was attached to the surface of wood nanofibers, and then iron ions were adsorbed to obtain the precursor, which was pyrolyzed to obtain Fe–N–CNFs, iron-nitrogen co-doped carbon nanofiber catalysts with encapsulated iron carbide particles [131]. In addition to the adsorption of inorganic iron ions, an organic iron source can be used instead (e.g., iron phthalocyanine) iron salts adsorbed on the cellulose surface [132], which facilitates the generation of catalysts with smaller particles of carbon layers encapsulated with iron carbide (Figure 15), and the ORR catalytic activity of this catalyst is further improved. This indicates that the dimension of iron carbide particles in such catalysts is closely connected to the catalytic activity of ORR, probably because, for one thing, larger particles affect the conductivity of the catalyst, and for another thing hand relatively uniform and fine particles can have a larger interfacial contact area, which facilitates the activation of the encapsulated carbon layer.

In addition to the aforementioned fibrous catalysts, iron carbide-modified lamellar carbon material electrocatalysts have also gained a lot of attention [133,134,135]. For example, Jiang et al. [133] prepared iron-nitrogen co-doped carbon nanosheet catalysts containing encapsulated iron carbide particles by pyrolysis of a mixture of urea, glucose, and iron salt precursors, and this bifunctional electrocatalyst exhibited efficient ORR and OER catalytic activities. Liu et al. [135] prepared an efficient sheet structured by pyrolysis of inexpensive precursor mixtures (melamine, iron salt, and o-phenanthroline) of iron carbide-modified doped carbon catalysts, which showed efficient OER catalytic activity in both basic and acidic media. Moreover, iron-based MOFs or COPs are often used to prepare such catalysts [136]. For example, MIL-88B served as the guest and ZIF-8 as the main body to synthesize the precursor of MOF-in-MOF structure [136]. This structure ensures in situ restricted pyrolysis of MIL-88B nanorods and easy preparation of nitrogen-doped carbon nanotubes with tiny Fe_3_C nanoparticles. The organic ligand-derived carbon matrix during the pyrolysis of this precursor effectively prevents the aggregation of the formed Fe_3_C nanoparticles. The resulting catalysts exhibit excellent catalytic activity for oxygen reduction in alkaline media due to a large number of tiny carbon-encapsulated Fe_3_C particles and the multilevel pore nitrogen-doped carbon structure. Kong et al. [137] reported porous nitrogen-doped Fe_3_C@Cs catalysts obtained by pyrolysis of covalent porphyrin polymers (CPP) generated from terephthalaldehyde, pyrrole, and iron chloride (Fe–CPP), in which the carbon encapsulated in the nitrogen The iron carbide particles encapsulated in the nitrogen-doped carbon shell have particle sizes ranging from 10–50 nm, and the catalyst exhibits efficient oxygen reduction catalytic activity.

For the synthesis of such encapsulated iron carbide particle catalysts, the application of certain organic iron sources facilitates the preparation of iron carbide nanoparticles with small particle sizes in the catalysts. Increasing the density of encapsulated iron carbide nanoparticles or introducing heteroatoms such as phosphorus into the carbon matrix can modify the catalyst structure to design and synthesize efficient electrocatalysts. Furthermore, hard or soft template methods can be used to fabricate multi-stage porous carbon materials to promote the mass transfer and electron transfer capability of catalysts.

According to the reported literature, carbon-coated iron carbide particle catalysts with various morphologies and structures can be prepared using hard or soft template methods. Guo et al. [138] synthesized iron carbide nanoparticles modified with bamboo nitrogen-doped carbon nanotubes using a mixture of melamine, P123, and Fe(NO_3_)_3_ by soft template induction method, where P123 acted as a soft template P123 acts as a soft template to induce the synthesis of carbon in one-dimensional direction. An amphiphilic colloidal template approach was devised by Li et al. to prepare catalysts with iron carbide encapsulated in mesoporous carbon spheres due to their unique structure leading to excellent ORR catalytic activity and stability [139]. Dopamine was used to synthesize polydopamine nanospheres, and then iron ions were adsorbed to obtain precursor Fe-PDA nanospheres. The precursor was pyrolyzed in a nitrogen atmosphere and then etched to eliminate the remaining silica, resulting in a catalyst Fe_3_C@mCN with ultra-small iron carbide particles and mesoporous nitrogen-doped carbon nanospheres. Tan et al. [140] used an asymmetric triblock copolymer (PS-b-P2VP-b-PEO) as a template to synthesize iron-nitrogen co-doped hollow carbon spheres with iron carbide nanoparticle modification. Melamine formaldehyde resin was utilized as the nitrogen and carbon source, and then the precursor micelle@M-FR spheres were adsorbed and combined with iron salts to obtain the catalyst Fe_3_C–Fe–N/C with excellent oxygen reduction catalytic activity after pyrolysis of this precursor. All these findings demonstrate indicate that the synergistic impact between the composition and morphological structure of the catalysts leads to the catalysts exhibiting better catalytic activity for oxygen reduction.

#### 3.2.2. Fe/Co Nanoparticles/Carbon Composite Electrocatalyst

**Figure 16:**
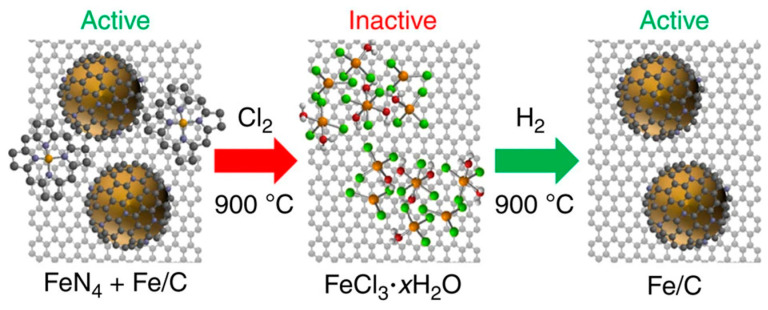
Effect of Cl_2_ and H_2_ treatments on Fe species and ORR activity [141].

The preparation of such catalysts by high-temperature pyrolysis generally has a more complex composition. Gewirth et al. [141] published an important study to reveal the true active center of such catalysts in the oxygen reduction reaction. The catalysts in their investigation included Fe–N species and iron nanoparticles enclosed by nitrogen-doped carbon layers. The catalysts were treated with chlorine and hydrogen at high temperatures, as illustrated in Figure 16, and the catalytic activity was found to be mainly derived from the nitrogen-doped carbon-layer-encapsulated iron nanoparticles rather than Fe–N species by passivating and activating the catalysts. Thus, it was demonstrated that such nitrogen-doped carbon layer-encapsulated Fe nanoparticles facilitated the efficient catalytic oxygen reduction reaction. Further, Strickland et al. [142] argued that the electron transfer from the Fe nanoparticles to the outer nitrogen-doped graphitic carbon is the main reason for the high-efficiency catalytic activity, suggesting that both Fe nanoparticles and nitrogen doping on the carbon layer are necessary active components.

Cobalt complexes are also often used to prepare such catalysts, such as the preparation of cobalt nanoparticles/carbon catalysts without Co–N species using N-heterocyclic carbene–Co complexes as precursors [143]. Based on the experimental results, it is found that the catalytic activity of the catalyst for oxygen reduction comes from the Cmurn site outside the cobalt nanoparticles. Further theoretical calculation shows that the electron transfer from the cobalt nanoparticles to the nitrogen-doped carbon layer leads to charge redistribution, thus, reducing the work function of the outer carbon surface. However, Li et al. [144] reported that the Co–N–C species in this kind of catalyst is the active site, while the internal cobalt particles have no catalytic activity for oxygen reduction. It has been revealed that FeCo alloy nanoparticles encapsulated in nitrogen-doped carbon have efficient catalytic activity for oxygen reduction as well [145,146]. Considering the commercial application, the stability of the catalyst and its ORR catalytic activity in the acidic medium is very important parameters. So as to promote the catalytic activity of this kind of catalyst in an acidic medium, other heteroatoms (B, P, or S) can be further introduced to adjust the charge distribution of the catalyst. Park et al. [147] synthesized nitrogen-sulfur co-doped carbon catalysts coated with iron nanoparticles in graphite carbon shell by hard template method. Because of the synergistic effect between coated iron nanoparticles and N, S co-doped carbon, the catalyst has excellent catalytic activity for oxygen reduction in an acidic medium. Different from the above-mentioned encapsulated catalysts, the catalyst still exhibits considerable activity in an acidic medium. It reveals that the addition of other heteroatoms to adjust the charge distribution on the doped carbon further is an effective method to improve the activity of the catalyst in the acidic medium. Moreover, to improve the stability of the catalyst, Mahmood et al. [148] used a kind of sandwich-like precursor, which introduced iron ions into a two-dimensional C_2_N matrix, and the catalyst obtained by pyrolysis had excellent stability.

**Figure 17:**
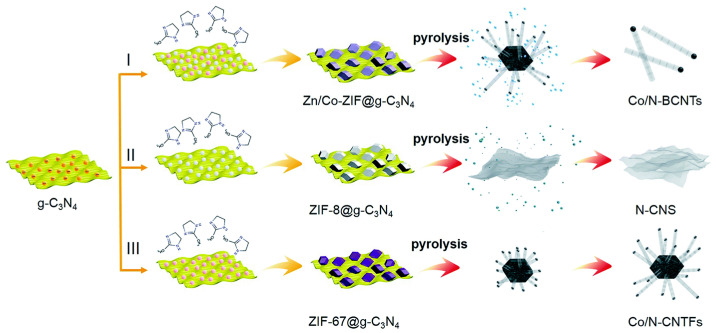
Diagram of the fabrication of nitrogen-doped carbon nanomaterials [152].

Metal framework organic compounds (MOFs) are widely used in the preparation of electrocatalysts because of their various types and ordered structures. Many kinds of encapsulated iron/cobalt nanoparticle electrocatalysts can be prepared by using MOFs as the precursor. MIL-100 is a kind of iron-based MOF, which is easy to form uniform iron nanoparticles when calcined at high temperatures, and then catalyzes the carbon atoms around it to form a graphite carbon layer. The prepared composite catalyst shows excellent catalytic performance for oxygen reduction [149]. ZIF-67 is a common cobalt-based MOF, which could be utilized as a precursor in the preparation of various cobalt-based carbon catalysts. For example, Dou et al. [150] added Co_3_O_4_ nanoparticles in the fabrication of ZIF-67 to obtain Co_3_O_4_@ZIF-67 and then pyrolyzed the precursor to prepare carbon nanotubes/porous carbon composite electrocatalysts with cobalt nanoparticles. The catalyst showed excellent ORR and OER catalytic activity, and the assembled zinc-air battery showed good activity and cycle stability. Li et al. [151] designed and synthesized nitrogen-doped carbon nanotubes modified by cobalt nanoparticles using ZIF-67. ZIF-67 was grown on the outer surface of halloysite nanotubes by electronegative adsorption of positive divalent cobalt ions on the outer surface of halloysite nanotubes. The catalyst with a high specific surface hollow nanotube structure was generated by pyrolysis and etching. In addition, mixed ZIF-67 (mixture of ZIF-8 and ZIF-67) is often used as a precursor for the preparation of catalysts; for example, different mixed ZIF-67 can be grown on nano-lamellar g-C3N4 by changing the proportion of Zn/Co in raw materials. It has been found that the resulting carbon material is directly associated with the ratio of Zn/Co [152]. In comparison to two-dimensional carbon nanolayers and three-dimensional carbon nanotube structures, one-dimensional bamboo-like nanotube structure carbon materials have efficient oxygen reduction catalytic activity (as illustrated in Figure 17), indicating the important role of carbon structure in catalytic activity. In addition, a highly efficient bifunctional catalyst with a tetragonal microstructure can be obtained by pyrolyzing the combination of cobalt salt and g-C_3_N_4_, which shows outstanding catalytic activity in oxygen reduction and oxygen evolution [153].

**Figure 18:**
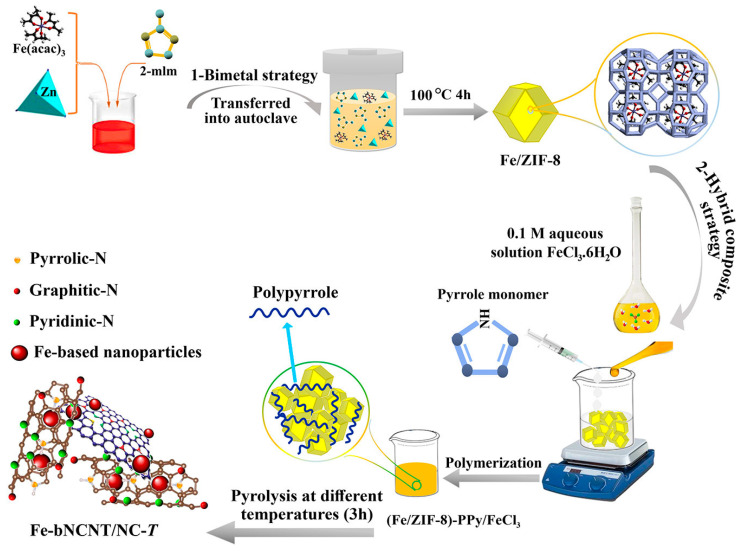
Schematic of the Fe/ZIF-8 Formation in the Bimetal Strategy (Step One), Then (Fe/ZIF-8)−PPy/FeCl3 Hybrid-Composite Strategy (Step Two), and Finally Obtained Catalysts via Thermal Treatment [154].

Very recently, Gharibi et al. [154] reported a new MOF-template assembly strategy for the preparation of iron-nanoparticle-loaded nitrogen-doped carbon nanotube/carbon sheet composites. To achieve the best ORR performance, this study combined two different approaches: bimetallic (Zn/Fe) MOF strategy and then hybridized the prepared MOF with pyrrole (Figure 18). The catalyst has high activity in both acidic and alkaline media.

Therefore, MOFs or some metal complexes are often utilized as precursors in the fabrication of this kind of catalyst. Most of these catalysts show good catalytic activity for oxygen reduction or oxygen precipitation in alkaline media. Further introduction of other heteroatom doping will make the catalysts produce more active sites, thus, improving their catalytic activity for oxygen reduction in acidic media. However, there is still a dearth of knowledge of the catalytic mechanism of such catalysts. On the one hand, it is debatable whether Fe/Co–N is a suitable species for catalytic ORR; on the other hand, there is a lack of research on the active sites of OER catalyzed by this kind of catalyst.

#### 3.2.3. Transition Metal Phosphide Nanoparticles/Carbon Composite Electrocatalyst

Transition metal (mainly iron, cobalt, and nickel) phosphide/carbon composite electrocatalysts are widely used in hydrogen evolution reaction (HER), oxygen evolution reaction (OER), and oxygen reduction reaction (ORR) [155,156]. During the preparation of catalysts by pyrolysis, precursors containing transition metals are prone to form metal nanoparticles, while heteroatoms are doped into the carbon material to form defective carbon. On the other hand, nanoparticles promote catalytic oxygen reduction reactions by forming synergistic interactions with the outer encapsulated carbon layer or active sites in the surrounding carbon matrix. Such composite catalysts as nanoparticles/carbon-doped can be divided into two categories: those containing bare nanoparticles and those containing graphitic carbon layers encapsulated with nanoparticles [123]. Due to the prolonged contact with the electrolyte solution, the bare nanoparticles are very susceptible to oxidation and dissolution, resulting in poor catalyst activity and stability, while the wrapped carbon layer can serve to protect the nanoparticles. Some studies have attributed the oxygen reduction activity to the synergistic effect of nanoparticles and the outer wrapped carbon layer, and further, some studies have found from the calculation results that there is electron transfer between nanoparticles and the wrapped carbon layer, which facilitates the oxygen reduction reaction. The morphology, structure, and composition of the composite catalysts were obtained by different precursors and different methods of synthesizing catalysts. The type, size, and distribution of nanoparticles are important factors affecting the electrocatalytic activity, and the pore structure and morphology of the carbon matrix are also important factors affecting the catalytic activity.

Although the transition metal phosphide itself has metal characteristics, the composite catalyst prepared by the combination of phosphide and carbon matrix shows excellent electrocatalytic activity to weaken the contact resistance and improve the conductivity of the catalyst. For example, the composite catalyst Ni_2_P/CNSs was obtained by loading nanostructured Ni_2_P onto carbon nanospheres. The results show that the carbon content of the catalyst influences HER activity. The outstanding catalytic activity and stability are owed to the large specific surface area and electrical conductivity of the catalyst and the synergism between Ni_2_P particles and carbon nanoparticles [157]. In addition, Ni_2_P particles were doped into N-doped graphene to generate a Ni_2_P/NRGO catalyst, in which a large number of Ni_2_P particles were evenly disseminated and synthesized in situ on nitrogen-doped graphene. The high conductivity and the synergistic effect between nickel phosphide nanoparticles and nitrogen-doped graphene are key factors in the high HER catalytic activity of the catalyst [158].

Recent studies have shown that cobalt phosphide-based catalysts are excellent multifunctional catalysts with high catalytic activity on HER, OER, and ORR. For illustration, it has been mentioned that the composite catalysts prepared by loading cobalt phosphide onto nitrogen-doped carbon nanotubes show remarkable OER and HER catalytic activity in 0.1 mol/L KOH solution [159]. A two-dimensional sandwich-like ZIF-67/GO precursor was prepared at room temperature, and the catalyst CoP/rGO-400 was obtained by pyrolysis and post-phosphating process [160]. Due to the porous structure, outstanding electrical conductivity, and the synergistic effect between CoP and graphene, the catalyst showed excellent OER and HER activity in alkaline media. Moreover, the specific surface area of cobalt phosphide particles was increased by introducing other metals to expose more active sites, hence improving the HER and OER catalytic activity of the catalyst. Liu et al. [161] prepared CoP/CNT catalyst by low-temperature phosphating precursor Co_3_O_4_/CNT. The catalyst shows outstanding HER activity and stability in acid electrolytes. In addition, the shell-core nano-cable catalyst CoP@C was formed using direct pyrolysis of the mixture precursors (Co (acac) ^2^ and PPh_3_) containing cobalt, phosphorus, and carbon. The cobalt phosphide nanorods in the catalyst are coated with a carbon layer to form a cable-like structure. The carbon layer not only improves the conductivity of the catalyst but also protects the internal cobalt phosphide nanorods from being dissolved, which makes the catalyst have brilliant HER catalytic activity and stability in an acidic medium [162]. The composite catalysts supported by metal phosphide on different carbon materials have also been further studied [163]. The HER catalytic activities of ordered mesoporous carbon modified by CoP particles, onion-shaped mesoporous carbon bubbles, macroporous carbon, or reduced graphene composites were tested, respectively. The findings indicate that the mesoporous structure and specific surface of the carbon matrix play an important role in the catalytic activity.

Besides CoP, Co_2_P is also a very efficient catalyst for OER and HER. Pan et al. [164] generated a variety of cobalt phosphide (CoP or Co_2_P) carbon composite catalysts. HER catalytic activity test shows that the activity order was CoP/NCNTs > Co_2_P/NCNTs, CoP/CNTs > Co_2_P/CNTs, and CoP > Co_2_P, indicating that the activity of CoP was higher than that of Co_2_P because the phosphorus content of CoP was higher than that of Co_2_P. In the process of hydrogen evolution, Co and P are both hydride and proton receptors and play significant roles in the reaction, so the best cobalt-phosphorus bonding benefits HER’s efficient catalytic activity. However, for the OER reaction, Co_2_P shows better catalytic activity than CoP, the content of Co in Co_2_P is higher than that of CoP, which leads to more exposure to active sites. On the other hand, because the OER reaction occurs in cobalt oxide/cobalt hydroxide and phosphate reconstruction on the surface of the catalyst, Co_2_P is more likely to form active sites, thus, promoting the OER reaction.

Chen et al. [165] reported that the electronic interaction between the encapsulated Co_2_P nanoparticles and the heteroatom-doped coated carbon layer makes the catalyst have efficient ORR catalytic activity. Heteroatom-doped mesoporous carbon nanotubes modified by Co_2_P nanoparticles were prepared by heating the mixture of cobalt acetate and organic-inorganic hybrid polymer nanotubes. The heteroatoms on the carbon layer break the electrical neutrality of the carbon matrix and facilitate oxygen adsorption. The internal Co_2_P nanoparticles promote the reduction of oxygen molecules on the carbon layer by giving electrons. Later, it was reported that the carbon catalysts modified by cobalt phosphide particles showed excellent catalytic activity in oxygen reduction [166,167,168]. Meng et al. [166] built CoP QDs embedded in a sulfur-nitrogen co-doped carbon catalyst. The strong force between organic phosphoric acid and cobalt ions was used in the synthesis process to ensure that CoP is small in size and not easy to agglomerate during pyrolysis. Wang et al. [167] fabricated hollow porous nitrogen-doped carbon rods modified by Co_2_P particles by ball milling. Jiang et al. [168] synthesized graphene modified by Co_2_P particles by supramolecular gel-assisted method to prevent particle agglomeration. However, the ORR catalytic activity of the above-mentioned catalysts in an alkaline medium is still lower than that of commercial Pt/C catalysts. Wang et al. [168] developed a unique fabrication to generate a CoPx particle-modified multi-stage porous tubular carbon catalyst. The catalyst showed excellent ORR catalytic activity and outperformed commercial Pt/C catalysts in alkaline electrolytes. In their study, CoPx nanoparticles and carbon nanotubes encapsulated in tubular carbon were obtained by using pyridine-modified carbon nanotubes, melamine, phytic acid, and porphyrin cobalt mixture precursors. This multi-stage carbon structure makes the catalyst have excellent electron transport and mass transfer properties, which contribute significantly to the catalyst’s ORR activity.

According to the reported studies, iron-based catalysts such as FeP generally show better ORR catalytic activity than cobalt-based catalysts [169]. Zhang et al. [170] used phytic acid and folic acid as phosphorus and nitrogen sources to synthesize nitrogen-phosphorus co-doped carbon nanosheet catalyst FeP@NPCs modified by FeP nanoparticles. Compared with single heteroatom doping, nitrogen and phosphorus co-doping results in more active sites on the surface of carbon nanosheets, and mesoporous catalyst FeP@NPCs show efficient ORR and OER double catalytic activity in alkaline electrolytes. Xu et al. [171] generated nitrogen-phosphorus co-doped carbon nanosheet catalysts modified by iron phosphide nanoparticles by pyrolysis of cheap precursors. The catalysts showed outstanding ORR catalytic activity and excellent stability in both acidic and alkaline electrolytes. After the removal of FeP nanoparticles by ball milling and pickling, the ORR catalytic activity of the catalyst decreased remarkably. Therefore, it can be proved that the synergism between iron phosphide nanoparticles and heteroatom-doped carbon makes the catalyst have efficient catalytic activity. Similarly, Hu et al. [172] prepared a nitrogen-phosphorus co-doped carbon nanosheet catalyst containing FexP nanoparticles, which showed high ORR catalytic activity in various PH electrolytes. The authors also proved that the synergism between FexP and nitrogen-phosphorus-doped carbon made the catalyst show high ORR catalytic activity.

### 3.3. Non-Previous Metal Porous Carbon-Based Monatomic Catalyst

One of the most important factors determining catalytic performance is the catalyst particle size; to achieve high specific activity and reduce cost, the size of catalyst particles must be reduced [173]. Monatomic catalysts are formed by anchoring a single non-precious metal atom in the matrix carbon [174,175]. Compared with nanoparticle-supported electrocatalysts, monatomic catalysts have the most surface-accessible atoms, which leads to greatly improved atomic utilization and catalytic activity [176]. Furthermore, the low-coordination environment of metal atoms and strong metal-carrier interactions are beneficial for catalytic stability and selectivity [177].

The study of monoatomic catalysts can be traced back to 1964 [178], Jasinski found that cobalt phthalocyanine had ORR catalytic activity under alkaline conditions, which proved the potential of macrocyclic compounds in electrocatalysis, but the activity and structural stability of macrocyclic compounds are poor under acidic conditions. In 1976, Jahnke et al. [179] subjected carbon-loaded macrocyclic compounds to pyrolysis to obtain carbon-loaded cobalt-based catalysts with significantly higher catalytic activity and stability under acidic conditions, which offered the possibility of replacing Pt-based metals in ORR with non-precious metal catalysts. In 1989, Yeager et al. [180] heat-treated the mixture of the carbon support, nitrogen-containing small molecules, and metal inorganic salts at high temperature, and obtained catalysts similar to macrocyclic compounds, which effectively avoided the problems of high price and poor stability, and provided more possibilities for the design of catalysts.

Among the transition metal-carbon catalysts, metal–nitrogen–carbon (M/N/C) catalysts have the most potential. Numerous research has demonstrated that catalysts with M-Nx sites formed by introducing N and non-precious metal elements (e.g., Fe, Co, Ni) into carbon exhibited excellent catalytic activity for oxygen reduction reactions [181,182,183]. The first discovery of the ORR catalytic activity of this structure was in metal macrocyclic compounds, such as phthalocyanines or porphyrins containing M-N_4_ centers. Therefore, for a long time, such compounds became a hot topic for improving their ORR catalytic activity by changing the macrocyclic structure and metal species, and this M-N_4_ center was considered the active site [183].

**Figure 19:**
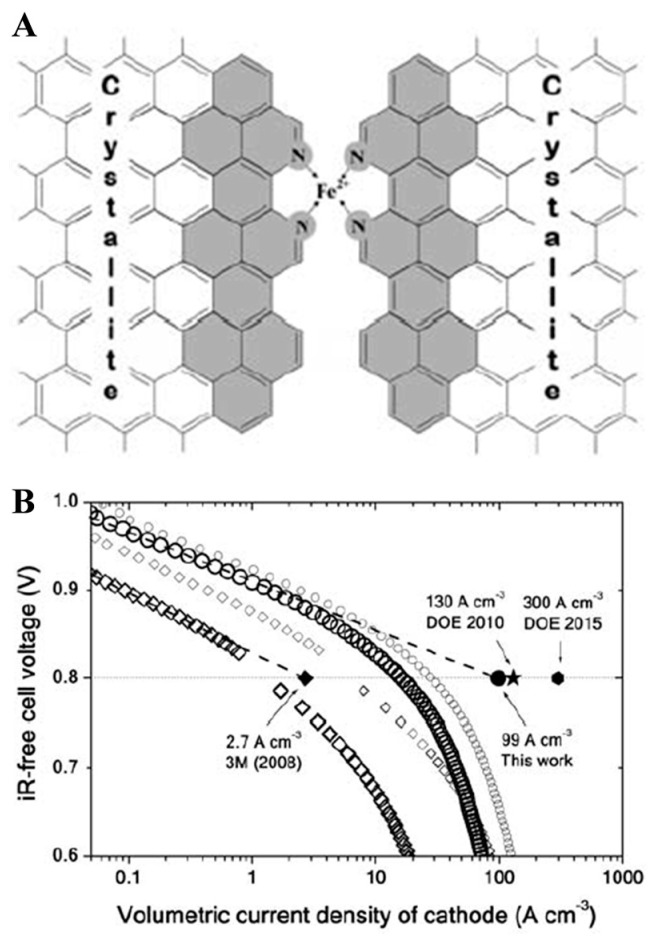
(**A**) Plan view of the catalytic site and graphitic sheet growth between two crystallites after pyrolysis, (**B**) Volumetric current density of best NPMC [186].

In recent years, there have been numerous studies on M/N/C [184,185]. The most representative one is published in Science by Dodelet’s group [186], in which the high specific surface carbon carrier Black Pearls 2000 was mixed with o-phenanthroline and ferrous acetate by sufficient ball milling and further ammonia treatment after high-temperature treatment to obtain Fe/N/C catalysts. Figure 19A shows the catalytic site between two crystallites after pyrolysis. The high-temperature carbonization of o-phenanthroline as a pore-filling agent formed an amorphous carbon material, and the ammonia treatment increased the nitrogen doping of the amorphous carbon material while increasing the microporous content of the carbon material and forming more Fe/N/C active centers. The current density of the catalyst was up to 99 A cm^−3^ at 0.8 V, as shown in Figure 19B.

The carbon materials used in the M/N/C are mostly commercial carbon black, which has the disadvantage that the porous structure is not easy to be adjusted. Therefore, researchers began to try to use porous carbon substrates to support single atoms.

In 2011 Dodelet’s team replaced BP-2000 with ZIF-8. The high nitrogen content and rich microporous structure of ZIF-8 laid a solid foundation for efficient ORR [187]. Compared to BP-2000, the addition of ZIF-8 improved the mass transfer of the catalyst and increased the bulk current density to 230 A cm^−3^, which is similar to the target value set by the US Department of Energy in 2015 (300 A cm^−3^).

**Figure 20:**
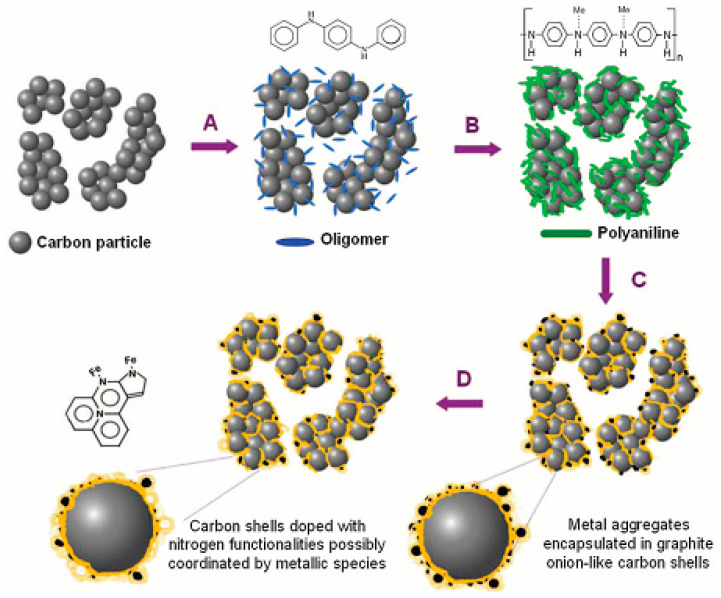
Schematic diagram of the preparation of M–N–C by pyrolysis of carbon precursors containing Fe and Co [188].

In the same year, Zelenay et al. [188] used short-chain aniline oligomers mixed with high specific surface area Ketjenblack carbon material (Ketjenblack EC-300J), cobalt nitrate, and ferric chloride metal salts, polymerized in the presence of oxidizer (NH_4_)2S_2_O_8_, and further carbonized at high temperature to obtain PANI-FeCo-C catalysts. The process of pyrolyzing carbon precursors containing Fe and Co under high temperatures is shown in Figure 20. The catalyst was used for ORR performance testing under acidic conditions, and its half-wave potential and Pt/C differed by 70 mV, while it had excellent stability performance.

ZIF is an ideal precursor to achieve homogenization of N M–N–C sites due to its special nN-containing ligands. ZIF-8-based catalysts have improved activity compared to carbon black. However, although ZIF-8 has a high specific surface, its pore structure is mostly microporous, which plays the role of active site loading in ORR but is not conducive to mass transfer. A sufficiently large pore structure can ensure the effective transfer of reactants to the whole electrode layer. In addition, effective charge transfer can ensure the full utilization of active sites and directly lead to the reduction of overpotential.

Liu et al. [189] adopted a mixture of polyacrylonitrile electrospun wire and ZIF-8, and the pore structure and FeNx ratios of the catalysts were adjusted by adjusting the additions of ZIF-8 and electrospun wire. The rich pore structure formed by the accumulation of carbon nanofibers makes Fe/N/CF exhibit excellent catalytic activity.

**Figure 21:**
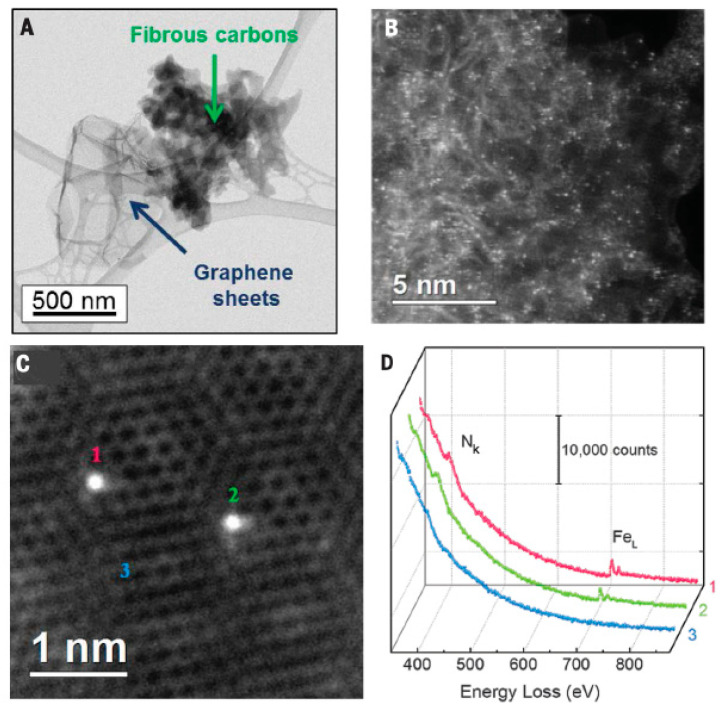
Morphology and structure characterization of the (CM + PANI)–Fe–C catalyst. (**A**) BF-STEM image; (**B**,**C**) HAADF-STEM image; (**D**) EEL spectra [190].

Zelenay et al. [190] prepared Fe/N/C catalysts with high porosity using Polyaniline, cyanamide, and metal salts. The atomic image of FeN_4_ was determined by an aberration-corrected scanning transmission electron microscope (Figure 21), and it was confirmed that FeN_4_ was the active site of ORR, which provided an important reference for future research. The porous structure of the catalyst not only improves the mass transfer of ORR but also facilitates the exposure of the active sites of the catalyst, which makes the catalyst have excellent ORR performance.

Research has confirmed that the M–Nx site is the active center for catalyzing ORR, but further study of the catalytic mechanism is still difficult. Taking Fe-based catalysts as an example, iron-based nanoparticles, such as Fe_3_C, Fe_3_N, Fe_3_O_4_, etc., will inevitably be produced during the high-temperature pyrolysis preparation process, resulting in complex components in the catalyst [191,192]. Current strategies to improve atomic dispersion include exploiting defect design, steric confinement, and coordinative anchoring.

Cheng et al. [193] used high-temperature pyrolysis to generate catalysts with single-atom iron content up to 7.7 ± 1.3 wt%, which are rich in Fe–N sites and have onset and half-wave potentials up to 0.950 V and 0.804 V, respectively, in acidic electrolytes, comparable to the activity of commercial Pt/C catalysts. The polymer electrolyte fuel cell assembled with this catalyst as a cathode catalyst exhibited a maximum power density of 325 mW cm^−2^ at 230 °C and maintained excellent stability at this temperature.

**Figure 22:**
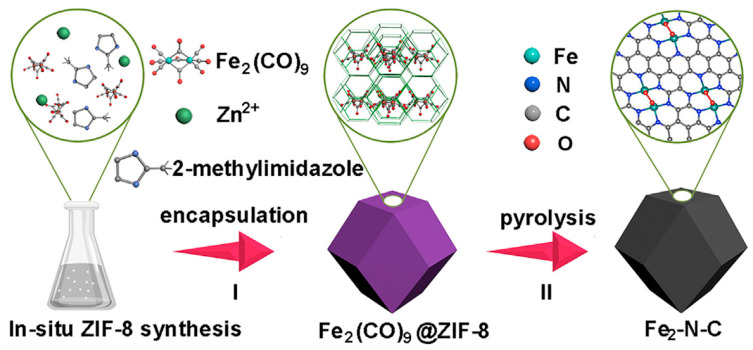
Schematic of the synthesis of Fe_x_–N–C [194].

Ye et al. [194] optimized the catalyst ORR catalytic activity by precisely tuning the number of iron atoms in Fe–Nx clusters. They synthesized Fe_1_–N–C, Fe_2_–N–C, and Fe_3_–N–C catalysts by introducing different iron salts into ZIF-8, respectively (Figure 22), where the different forms of oxygen molecule adsorption on different structural iron centers caused different energies required for oxygen-oxygen bond breakage. The catalyst Fe_2_–N–C was shown to have the greatest ORR catalytic activity, with a half-wave potential of up to 0.78 V measured in 0.5 mol/L H_2_SO_4_ solution, and also exhibited excellent stability.

In addition to the most commonly used Fe-based catalysts, other non-precious metal monoatoms have also been used for oxygen reduction reactions, such as MnNx and CuNx, all of which have excellent catalytic effects. As an illustration, Li et al. [195] investigated a method for a greater yield of Cu monatomic catalysts. In this method, the bulk copper metal is oxidized into volatile Cu(NH_3_)x by NH_3_, which is trapped by nitrogen-rich porous carbon defects to form a monoatomic Cu-based catalyst. Specifically, the method is first based on the strong Lewis acid-base coordination effect, which aligns ammonia with copper atoms to form a volatile Cu(NH_3_)x substance; then, under an ammonia atmosphere, Cu(NH_3_)x is trapped by nitrogen-doped carbon defects to form a monatomic copper catalyst. This method can be suitable for preparing industrial-grade nickel and cobalt-based monatomic catalysts.

Recent studies have shown that loading bimetal on N-doped carbon can further improve the electrocatalytic activity [196,197,198]. Compared with monometallic sites, bimetallic monoatoms have a synergistic effect, which creates more favorable sites for bond breaking in O_2_ in the ORR process, thus, showing enhanced properties.

**Figure 23:**
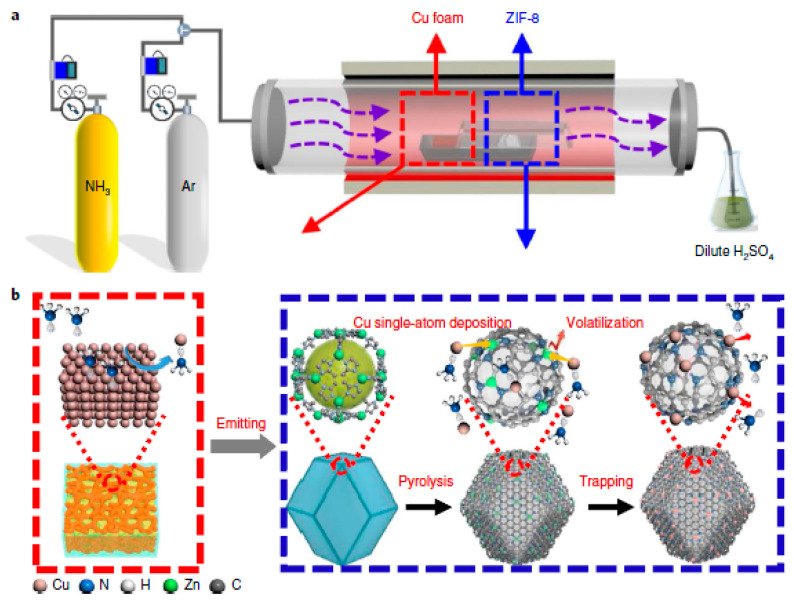
Diagram of the fabrication method of Cu–SAs/N–C. (**a**) Apparatus diagram; (**b**) Proposed reaction mechanism [195].

Li et al. [199] constructed a novel Fe–Co bisite electrocatalyst loaded with N-doped carbon nanotubes to precisely control the bonding between the Fe^3+^ precursor and the Co node of the Zn/Co bimetallic organic skeleton. The catalyst preparation process and device are shown in Figure 23. The encapsulated guest metals Fe and Co produced Fe–Co bimetallic sites that were adsorbed in the cavity of the bimetallic organic skeleton. After high-temperature carbonization, the FeCo sites on the surface of the bimetallic organic skeleton catalyzed the growth of carbon nanotubes, and the bimetallic organic skeleton was transformed into an N-doped carbon nanotube-loaded bimetallic site catalyst ((Fe, Co)/CNT). The starting potential (1.05 V) and half-wave potential (0.842 V) are far superior to those of the Pt/C electrode. The catalyst was employed as a cathode catalyst in Zn–Air batteries and exhibited high discharge current density, with current density and specific energy density reaching 260 mW cm^−2^ and 870 Wh kg^−1^, respectively.

Ma et al. [200] developed a self-assembly method for the stepwise synthesis of Ni and Fe dual monatomic catalysts. Ni monatoms located on the inner wall of graphitized hollow carbon nanospheres and Fe monatoms on the outer wall of graphitized hollow carbon nanospheres, the functions of the two metal single atoms are separated. Both Ni and Fe monatoms are tetra-coordinated with nitrogen elements, i.e., forming Ni–N_4_ and Fe–N_4_ planar structures, and the catalysts are noted as Ni–N4/GHSs/Fe–N_4_, which exhibit outstanding bifunctional electrocatalytic performance, where the outer wall Fe-N_4_ favors highly active ORR and the inner wall Ni–N_4_ favors highly active OER. Density flooding theory calculations indicate that Fe–N_4_ and Ni–N_4_ are the active sources of ORR and OER, respectively. Ni–N_4_/GHSs/Fe–N_4_ as cathode catalysts for Zn–Air batteries exhibit excellent cell energy efficiency and cycling stability, which are better than the performance of standard Pt/C+RuO_2_ electrode materials.

In addition to bimetallic sites, the introduction of other heteroatoms into the catalyst is also an effective way to regulate the catalytic performance. For example, the catalysts obtained by introducing heteroatomic sulfur into Fe/N/C catalysts showed more efficient ORR catalytic activity [201]. It is probably due to the different atomic radii and electronegativity of sulfur and nitrogen that sulfur doping may induce more defects on the carbon substrate on the one hand and affect the electron cloud distribution of surrounding atoms on the other hand. Unlike nitrogen, sulfur does not form coordination species with iron, which allows the catalyst to retain the Fe–Nx active center and only serves to modify the electronic structure further. Several studies have confirmed the existence of a synergistic interaction between doped sulfur and Fe–Nx active sites [202], and this synergistic mechanism is more evident in acidic and basic media due to the different reaction histories of oxygen reduction reactions in acidic and basic media.

Despite the remarkable catalytic performance of non-previous metal monoatomic electrocatalysts, their catalytic mechanism remains unclear. Building models or applying theoretical calculations to study the mechanism of monoatomic catalysis is a must-see topic. Wu et al. [199] coated the metal organic skeleton with surfactant F127 and heat-treated it to obtain the monatomic CoN–C catalyst with a core-shell structure. The core of the catalyst is Co–N–C porous carbon dispersed by atoms derived from ZIF, and the shell is a graphite carbon layer formed by the carbonization of F127. The domain-limiting effect of the outer surfactant inhibits the agglomeration of Co atoms and the collapse of the microporous structure in ZIF. In addition, after heat treatment, the limiting effect of the outer surfactant led to a higher nitrogen doping and active site density to maximize the catalytic performance. Co–N–C@F127 catalyst with a half-wave potential of up to 0.84 V (vs. RHE) under acidic conditions. Density flooding theory calculations show that, unlike other Co-based catalysts, this atomically dispersed Co–N–C@F127 catalyst contains plenty of CoN_2+2_ sites that are active and thermodynamically stable, favoring the occurrence of four-electron transfer in the ORR. This study illustrates the importance of constructing models to explore the activity and selectivity of atomic-scale catalysts for catalytic reactions and adjusting the atomic ratio and coordination environment can optimize the ORR performance.

However, despite the excellent reaction performance of non-precious metal monatomic carbon-based catalysts, there are still great challenges in their synthesis. Stability is a key factor restricting the application of monatomic catalysts. It is well known that the surface energy of a single atom is higher than that of clusters and nanoparticles, so a single atom tends to form aggregates. To maintain the atomic dispersion, the catalyst loading is kept at a low level at present. Therefore, increasing the loading capacity of single atoms is a challenge that must be faced. More in-depth understanding of the stabilization mechanism of monatomic catalysts is needed to lay a foundation for the practical application of monoatomic catalysts.

### 3.4. Performance of Carbon-Based Non-Precious Metal Electrocatalysts in Fuel Cells

At present, membrane electrode assembly (MEA) is often used to evaluate ORR catalysts, aiming to realize practical applications in fuel cells [203]. Currently, the only applicable cathode catalysis is the Pt/C catalyst, which is also the only catalyst that can meet the requirements of the US Department of Energy (DOE) [204]. For the catalysts practically used in fuel cells, DOE has set an activity and stability target for 2025, that is, (i) ORR activity must reach MA for 0.44 Amg^−1^ _Pt_ at 0.9 V_iR-free_, (ii) stability must reach <40% MA loss after 30,000 cycles.

With the approach of the commercialization of fuel cell technology, developing highly active, low-cost non-precious metal electrocatalysts to replace the currently used platinum-based catalysts is necessary to reduce costs and ensure large-scale applications [205].

Up to now, the activity of many reported transition metal carbon-based catalysts is comparable to that of platinum-based catalysts. The HER performance of CoMoP@C materials proposed in 2017 is close to that of commercial 20% Pt/C under the condition of pH = 0–1 and even exceeds it when pH = 2–14 (for example, η > 240mV, pH = 2.2) [206].

Carbon-supported transition metal nitrogen-containing complexes (M–Nx/C) are considered to be the most promising ORR catalysts, which exhibit similar activity and stability to commercial Pt/C catalysts [207]. Through the synergistic action of the local structure of N coordination, the adsorption energy of the active site for oxygen-containing intermediates is lower [208]. In a recent study, Fe–Nx–C catalysts have shown a current density of 1.55 A cm^−2^ at an operating voltage of 0.43 V, which is a good performance among similar catalysts. However, its stability still needs to be further improved before practical application [209]. Co–N–C-based catalysts demonstrate a current density of 0.022 A cm^−2^ at 0.9 V_iR-free_ [210]. In 2022, Lilloja et al. [211]. Prepared transition metal- and nitrogen-doped mesoporous carbons. Among them, iron-containing materials exhibit impressive ORR activity, which is similar to that of commercial Pt/C. In 2021, Wang et al. [212] prepared Fe–N–C monatomic catalyst by gel restriction strategy, which prevented the agglomeration of Fe in the carbonization process and achieved high dispersion and small size Fe–N on the active site. The resulting Fe–SASC provides higher limiting diffusion current, as well as more positive initial potential and half-wave potential (E_onset_ = 1.00 V, E_1/2_ = 0.87 V), and has excellent methanol resistance (E_onset_ = 0.97 V, E_1/2_ = 0.85 V) compared with commercial Pt/C.

Although many non-noble metal carbon-based catalysts have shown high activity in the study, they cannot maintain the expected performance under practical operating conditions. The mainstream of fuel cell technology is acidic polymer electrolyte membrane fuel cells, but most of the reported ORR studies were carried out in KOH solution. Compared with that in alkaline electrolytes, the stability of metal nanoparticles in the acidic electrolyte is poor [213]. Although the design of carbon carriers or the preparation of monatomic catalysts can improve this, there is still a long way to go to meet the standards of the U.S. Department of Energy.

The problems of non-noble metal carbon-based catalysts mainly lie in the limited number of active sites, oxidation of the carbon substrate, and metal dissolution. Compared with platinum-based catalysts, the active sites of non-noble metal-based carbon-based catalysts are tightly bound to the carbon matrix, making it difficult to gain a clear understanding of the reaction mechanism.

In terms of avoiding the leaching of metal species under acidic conditions, metal-free carbon-based catalysts show great potential [214]. For example, N-doped graphene has good conductivity and mass transfer ability due to its huge specific surface area and layered structure, which ensures its ORR activity [215]. At present, N-doped graphene can catalyze oxygen reduction as efficiently as the state-of-the-art Fe–N–C catalyst, and its stability is better [216]. N-doped carbon nanomaterials have been reported as durable catalysts for oxygen reduction reactions in acidic fuel cells [217]. The carbon electrode without metal nanoparticles will not have obvious acid corrosion, so excellent stability has been observed in the fuel cell. The performance (initial potential and half-wave potential) of the phosphorus-doped carbon catalyst prepared by Liu et al. is better than that of commercial Pt/C under alkaline conditions [218]. The limited diffusion current density of Pt/C decreased by 31.4% after 12000 cyclic voltammetry scans, while the catalyst P-C had almost no attenuation, and its resistance to methanol poisoning was much higher than that of Pt/C.

Although great progress has been made in this field, the ORR activity and stability of non-noble metal catalysts remain challenging. In the research work, the ORR activity test is generally carried out at low current density or low power level, and its life is much lower than the target set by DOE. Therefore, it is still a long-term goal to improve the ORR activity and stability of non-precious metal carbon-based catalysts.

## 4. Summary and Outlook

Currently, the most widely used electrocatalysts are previous metal-based catalysts represented by Pt. Despite the satisfactory activity of these catalysts, their high cost and poor durability make it difficult to achieve large-scale applications.

Non-precious metal-based electrocatalysts are considered to be the hope of promoting the commercialization of electrochemical energy in the future, but the current situation of non-precious metal-based electrocatalysts still has a long way to go for large-scale application. This paper reviews the research results of non-previous metal porous carbon-based catalysts, mainly including metal-free carbon-based catalysts, non-previous metal monatomic carbon-based catalysts, and non-precious metal nanoparticle carbon-based catalysts. Undoubtedly, carbon-based non-noble metal electrocatalysts can be used as a promising electrocatalyst to replace Pt. However, there are still many issues that exist in this field and need to be studied in the future:

1. Non-previous metal porous carbon-based monatomic catalyst has an atomic utilization ratio close to 100%, which is a worthy research direction. However, its current performance cannot be compared with that of commonly used Pt/C catalysts. It is still necessary to develop more advanced synthetic methods to improve the stability of non-noble metal carbon-based monatomic catalysts and conduct more in-depth research on their structure-activity relationship to guide the synthesis of catalysts and improve their catalytic activity.
2. As an alternative to previous metal-based electrocatalysts, the large-scale production of non-previous metal porous carbon-based catalysts must be investigated. However, as far as the current technology is concerned, the large-scale preparation of non-precious metal electrocatalysts cannot be realized. Finding industrial-scale scale-up synthesis methods while maintaining low cost is the key to the practical application of non-precious metal catalysts. In addition, the uniformity of products should be ensured while mass production, which is a very challenging research direction in the future.
3. Porous carbon supports can improve the dispersion of active components and provide channels for material and charge transport. Therefore, carbon support is a key factor in the performance of the catalyst. However, the traditional carbon matrix will be corroded under the battery working conditions and lose the metal-support interaction. Therefore, the improvement of the carbon matrix is an important research direction. The design of porous carbon matrix channels can achieve a more uniform catalyst load, expose more active sites, and promote the mass transfer of reactants, thus, improving catalytic activity.
4. Many aspects are still poorly understood, such as the molecular-level description of solvents, cations, and anions near the electrode–electrolyte interface. On the basis of experimental research, using theoretical simulation to optimize the synthesis conditions and deduce the structure-activity relationship will be conducive to a more in-depth understanding.

## Figures and Tables

**Table 1:** Summary of the template, BET surface area, pore size, and carbonization conditions for diverse porous carbons by a hard template.

Sample	Template	BET Surface Area/m^2^ g^−1^	Pore Size/nm	Carbonization Conditions	Ref.
YTC8	Zeolite Y	1544	1.9	5 °C/min, 800 °C, 4 h	[41]
CEMC900	Zeolite EMC-2	3360	1.5	900 °C, 3 h	[42]
M-OMC	SBA-15	643	2.9	1.58 °C/min, 700 °C, 3 h	[47]
N-MC	SBA-15	535.2	4.15	3 °C/min, 600 °C, 5 h	[48]
CMK-5	SBA-15	2 84	4.1	4 °C/min, 850 °C, 1 h	[49]
CMK8-SC	KIT-6	740	5.0	1.4 °C/min, 900 °C, 2 h	[50]
OMC	KIT-6	905	4.0	900 °C, 3 h	[51]
Carbon	MgO	1290	0.58	10 °C/min, 900 °C, 1 h	[56]
GZnC	ZnO	2412	2–10	10 °C/min, 900 °C, 2 h	[57]

**Table 2:** The reported nitrogen-doped carbon electrocatalyst.

Sample	Electrolyte	Onset Potential	Electron Transfer Number	Ref.
N-doped hollow mesoporous carbon spheres	0.1 M KOH	0.92 V	3.99 @ 0.4 V	[89]
Nitrogen-doped graphite nanomaterial	0.1 M KOH	1.52 ± 0.02 V	-	[90]
Graphene/nitrogen-doped porous carbon sandwich	0.1 M KOH	0.99 V	-	[91]
nitrogen-doped carbon	0.1 M KOH	0.74 V	2.5–2.6 @ 0.1–0.5 V	[92]
3D porous N-doped graphene foam	0.1 M KOH	1.02 V	3.9 @ 0–0.85 V	[93]
N-doped nanoporous carbon nanosheet	0.1 M KOH	0.73 V	3.85–3.96 @ 0.06–0.9 V	[94]
**Sample**	**Electrolyte**	**Half-Wave Potential**	**Electron Transfer Number**	**Ref.**
Flaky N-doped carbonaceous material	0.1 M KOH	0.75 V	3.47 @ 1.2 V	[95]
Rod-like N-doped carbonaceous material	0.1 M KOH	0.76 V	3.74 @ 1.2 V	[95]
Nitrogen-doped carbon	0.1 M KOH	0.88 V	-	[96]
Nitrogen-doped carbon nanofiber aerogel	0.1 M KOH	0.80 V	3.96 @ 0.8 V	[97]

## Data Availability

Data available upon request.
